# Identification of *cis*-acting determinants mediating the unconventional secretion of tau

**DOI:** 10.1038/s41598-021-92433-3

**Published:** 2021-06-21

**Authors:** Taxiarchis Katsinelos, William A. McEwan, Thomas R. Jahn, Walter Nickel

**Affiliations:** 1 Heidelberg University Biochemistry Center (BZH), INF 328, 69120 Heidelberg, Germany; 2 Schaller Research Group, DKFZ, Proteostasis in Neurodegenerative Disease (B180), University of Heidelberg, INF 581, 69120 Heidelberg, Germany; 3 Department of Clinical Neurosciences, UK Dementia Research Institute, University of Cambridge, Cambridge, UK

**Keywords:** Protein transport, Alzheimer's disease, Secretion

## Abstract

The deposition of tau aggregates throughout the brain is a pathological characteristic within a group of neurodegenerative diseases collectively termed tauopathies, which includes Alzheimer’s disease. While recent findings suggest the involvement of unconventional secretory pathways driving tau into the extracellular space and mediating the propagation of the disease-associated pathology, many of the mechanistic details governing this process remain elusive. In the current study, we provide an in-depth characterization of the unconventional secretory pathway of tau and identify novel molecular determinants that are required for this process. Here, using *Drosophila* models of tauopathy, we correlate the hyperphosphorylation and aggregation state of tau with the disease-related neurotoxicity. These newly established systems recapitulate all the previously identified hallmarks of tau secretion, including the contribution of tau hyperphosphorylation as well as the requirement for PI(4,5)P_2_ triggering the direct translocation of tau. Using a series of cellular assays, we demonstrate that both the sulfated proteoglycans on the cell surface and the correct orientation of the protein at the inner plasma membrane leaflet are critical determinants of this process. Finally, we identify two cysteine residues within the microtubule binding repeat domain as novel *cis*-elements that are important for both unconventional secretion and trans-cellular propagation of tau.

## Introduction

A major pathological feature of several neurodegenerative diseases, including Alzheimer’s disease (AD), is the accumulation of hyperphosphorylated and detergent-insoluble tau assemblies^1^. Physiologically, tau stabilizes and promotes the assembly of microtubules^2^, a function mediated by the microtubule binding repeat domain (MTBR) and its flanking regions at the C-terminus^2,3^. However, under pathological conditions tau becomes extensively phosphorylated at epitopes normally unaffected in healthy individuals^4,5^. These series of modifications subsequently lead to its detachment from microtubules and contribute to the formation of intracellular aggregates, such as neurofibrillary tangles and paired helical filaments in AD^6^.

An important aspect in the development of the pathology is that the deposition of tau aggregates throughout the brain follows a stereotypic pattern, which directly correlates the affected regions with the occurring symptoms^7,8^. Moreover, the increased levels of extracellular tau in the cerebrospinal fluid (CSF) is a commonly used biomarker for diagnosis and staging of the disease, as they typically precede the onset of symptoms^9,10^. Therefore, over recent years the emerging hypothesis in the field includes the trans-cellular spreading of aggregated tau species, where pathological species spread from affected brain areas to healthy neuronal clusters and convert naïve tau to the pathological form^11^. Such processes have been experimentally reproduced in vivo and *in cellulo* through the administration of recombinant or ex vivo-derived fibrils, where in both cases the endogenous tau was effectively seeded into insoluble aggregates^12–15^.

We and others have demonstrated that the release of tau molecules from cells occurs via active cellular mechanisms that significantly impact the propagation of pathological conformations^16–18^. This set of protein externalization mechanisms are collectively termed Unconventional Protein Secretion (UPS) and the common feature within proteins following such routes is that their export to the exterior does not rely on the classical secretory pathway^19^. Initial reports proposed tau secretion to occur through a Type III UPS pathway mediated by exosomes^19–21^. However, the levels of tau associated with these vesicles are rather low when compared to the free tau protein, which represents about 90% of extracellular tau^22,23^. Recently, evidence for unconventional secretion of tau through a Type I UPS pathway has been reported^16,17^, a mechanism that is based on direct protein translocation across the plasma membrane^19,24^. More specifically in this process, the disease-associated hyperphosphorylation of tau reduces its affinity to the microtubules, leading to increased levels of intracellular protein available for secretion^16,25^. Subsequently, free cytosolic tau is recruited at the inner plasma membrane leaflet through its interaction with PI(4,5)P_2_^16^ as well as with other lipidic components, such as cholesterol and sphingomyelin^17^. Finally, the translocation process is completed by sulfated proteoglycans (PGs) on the outer cell surface, which act as anchor points that retain the majority of the extracellular tau population^16,26^.

This mechanism shares striking similarities with the secretory route of fibroblast growth factor 2 (FGF2), a leaderless signalling protein with significant endocrine and autocrine functions in development and tumorigenesis^19,24^. The efficient secretion of FGF2 is initiated by the docking of the protein to the Na^+^, K^+^-ATPase^27^ and the subsequent interaction with PI(4,5)P_2_ at the inner leaflet of the plasma membrane^28–30^. These two cellular components alongside Tec kinase^31^ and heparan sulfate proteoglycans (HSPGs)^32,33^ comprise the *trans*-elements that mediate unconventional secretion of FGF2. Concomitantly, the *cis*-elements include the positively charged epitopes of FGF2 mediating the interaction with Na^+^, K^+^-ATPase (K54 and K60)^34^, PI(4,5)P_2_ and HSPGs (K127, R128, and K133)^29^ as well as the phosphorylation target of Tec kinase (Y81)^31^. This set is completed with the two surface cysteine residues (C77 and C95) that foster the assembly of membrane-spanning FGF2-oligomers through the formation of intermolecular disulfide bridges^35^. Intriguingly, depending on the isoform type derived from the alternative mRNA splicing of exon 10, there are up to two cysteine residues in the amino acid sequence of tau (C291 and C322)^36^. As previous studies suggest that these residues are implicated in the formation of oligomeric species via inter- and intra-molecular disulfide bonds^37^, we questioned their potential contribution to the secretion of tau from cells.

In the current study, using in vivo and *in cellulo* systems, we correlate the phosphorylation status of tau with its aggregation propensity and the associated toxicity. Moreover, we demonstrate that secretion of tau to the extracellular space follows an active route, which is not significantly influenced by cell death, but requires the functional orientation of the protein at the inner plasma membrane leaflet for its successful translocation into the extracellular space. Most importantly, we identify a novel *cis*-element, namely the two cysteines within the MTBR that govern the unconventional secretion and trans-cellular propagation of tau to adjacent cells.

## Results

### Phosphorylation of tau determines its toxicity and secretion in *Drosophila melanogaster* models

**Figure 1:**
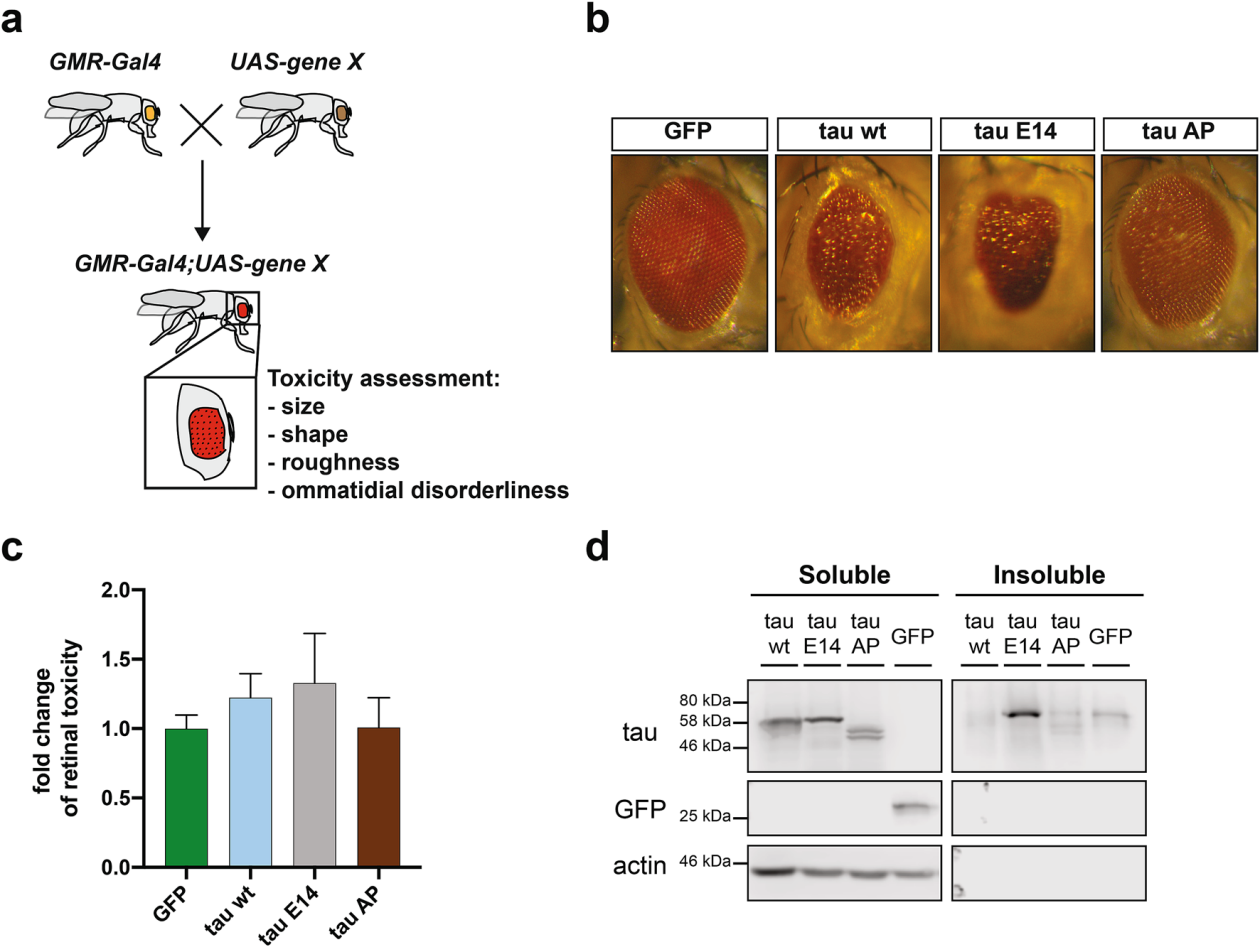
Tau hyperphosphorylation induces toxicity to *Drosophila melanogaster* upon expression in the retina. **(a)** Schematic illustration of the experimental setup and the parameters used to assess the toxicity in *Drosophila* retinal cells. **(b)** Representative images from *Drosophila melanogaster* eye phenotypes upon expression of different constructs under the *GMR-Gal4* promoter element. **(c)** The severity of roughness in the eye phenotype was quantified using the Flynotyper ImageJ-based plugin and then normalized to the GFP control. Bars represent mean values ± s.d., n ≥ 6 animals/genotype were assessed. **(d)** Sarkosyl-soluble and insoluble fractions were isolated from transgenic *Drosophila melanogaster* brain homogenates and blotted against pan-tau and GFP, while actin was used as loading and assay quality control.

The expression of human tau leads to a robust reduction in the lifespan of *Drosophila melanogaster*, which is further exacerbated by disease-associated mutations^38,39^. In order to delineate the impact of aggregation and phosphorylation on the in vivo-induced toxicity, we employed wild type (wt) human 0N4R tau and two variants bearing mutations in 14 commonly phosphorylated serine/threonine (S/T) residues^40,41^. In one construct, those S/T residues have been mutated to glutamate (E14) thereby mimicking the phosphorylated state. Alternatively, mutation of these S/T residues to alanine (AP) lead to complete impairment of phosphorylation at these sites. As the pan-neuronal expression of tau E14 under the *elav-Gal4* driver was severely toxic in our experiments during the developmental stages, we used a driver line for targeted integration and thereby equal expression of the different tau variants in neuronal and non-neuronal post-mitotic cells of the retinal tissue (*GMR-Gal4*, Fig. 1a). The major advantage of this system is that the eye-related toxicity does not affect animal viability^42^ and indeed we observed no impairment in the lifespan of these transgenic flies. In line with previous observations^43^, mutation of these S/T epitopes to a phosphomimetic state (E14) led to significantly enhanced toxicity compared to the AP variant or the control line expressing GFP (Fig. 1b,c). Biochemical analysis of these flies demonstrated that the tau E14 variant was substantially enriched in the Sarkosyl-insoluble fraction (Fig. 1d), suggesting that the presence of aggregated species contributes to the in vivo toxicity.

To allow a detailed analysis of the relationship between tau phosphorylation, aggregation and secretion propensity in a closely related experimental setup, we employed a neuronal cell culture line derived from the central nervous system of the fruit fly (BG2-c6)^44^ and ectopically expressed the aforementioned human tau variants. Initially, we assessed the expression characteristics of the wt variant in these cells under the metallothionein inducible promoter^45^. We biochemically confirmed that a C-terminally tagged version of tau with three repeats of hemagglutinin peptide (3xHA) was efficiently phosphorylated by endogenous kinases (Fig. S1a), indicating the functionality of human tau in the *Drosophila* neuronal cells. We next determined the Cu^2+^ concentrations and timings that allowed us to obtain optimal tau expression levels (Fig. S1b, c). Even though the intracellular accumulation of tau wt plateaued at about 60 h upon addition of Cu^2+^ (Fig. S1c), its phosphorylation was already apparent even at the earliest time points (Fig. S1d).

**Figure 2:**
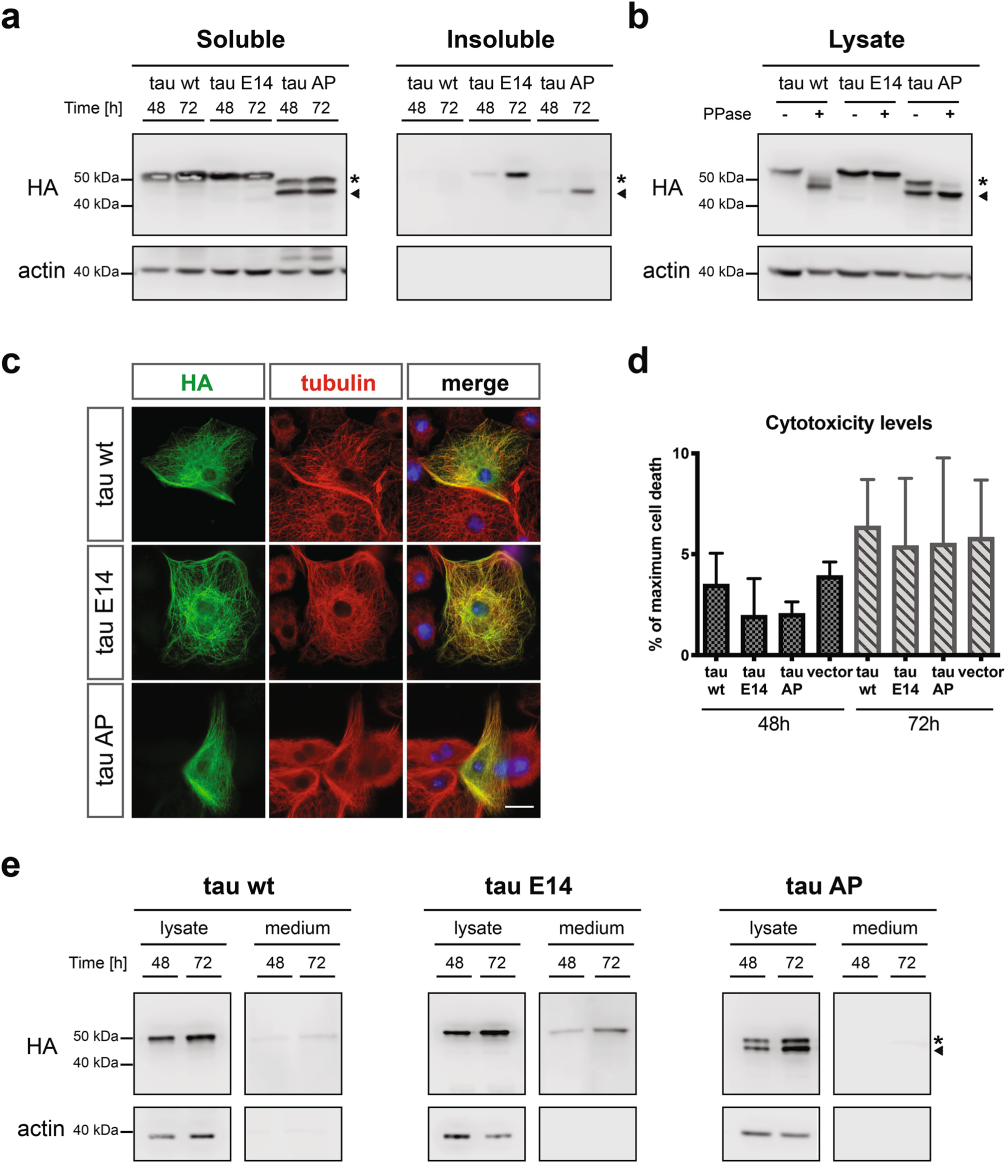
Phosphomimetic tau is preferentially secreted from *Drosophila* neuronal cells. **(a)** Sarkosyl-soluble and insoluble fractions from cells expressing the different tau variants were isolated and subsequently blotted against HA and actin. Asterisk and arrow indications correspond to the P- and non-P band of tau AP, respectively. **(b)** Lysates from transfected cells were subjected to dephosphorylation and then immunoblotted against HA. Actin was used as loading control. Asterisk and arrow indications correspond to the P- and non-P band of tau AP, respectively. **(c)** Antibodies against HA and tubulin were used to stain tau and tubulin, respectively, whereas the nucleus was stained with Hoechst (scale bar, 15 µm). **(d)** Conditioned media from cells expressing the different tau variants or the empty vector were collected and tested for levels of cell death, as determined by the release of LDH. Data represent mean values ± s.d., n = 3 biological replicates. **(e)** Lysates and immunoprecipitated medium from cells expressing the different tau variants were immunoblotted against HA for detection of tau and actin as loading and quality control. Asterisk and arrow indications correspond to the P- and non-P band of tau AP, respectively.

Having established this novel cell culture setup, we expressed and characterized wild-type tau alongside the two phospho-tau variants, E14 and AP. All constructs accumulated over time in the soluble fraction, with the tau E14 forming higher levels of Sarkosyl-insoluble aggregates compared to tau wt and tau AP (Fig. 2a). We also noted that the introduced mutations as well as the phosphorylation modifications led to characteristically distinct mobility in SDS-PAGE (Fig. 2a). Interestingly, the expressed tau AP variant in the *Drosophila* neuronal cells displayed two distinct protein bands, with only the lower molecular weight species being detected in the insoluble fraction (Fig. 2a). The difference in the SDS-PAGE mobility between these two bands was a result of differential phosphorylation by endogenous kinases, which we determined by in vitro dephosphorylation experiments (Fig. 2b). Moreover, our detailed phospho-mass spectrometry mapping (Fig. S1e, Supplementary Tables S1, S2, and S3) identified 4 residues that are exclusively phosphorylated in the high (high P-band, marked with an asterisk in Fig. 2a,b, and Fig. S1e), but not in the lower molecular weight versions of the tau AP variant (low P-band, marked with an arrow in Fig. 2a,b, and Fig. S1e). Nevertheless, all the tested tau variants demonstrated the expected cellular association with the cytoskeleton and more specifically at the microtubules (Fig. 2c), while in contrast to the in vivo data (Fig. 1a) none of them caused any apparent toxicity to the cells (Fig. 2d). As we have previously shown that phosphorylation acts as a pivotal driver of tau secretion^16^, we addressed this property in our new cell culture system. Having determined the optimal immunoprecipitation conditions to enrich for tau from the medium of the *Drosophila* neuronal cells (Fig. S1f), we tested the culture supernatants for the presence of each variant in different time points. Interestingly, the phosphorylation status of the tested tau variants correlated well with their secretion propensity, meaning that the phosphomimetic tau E14 was secreted already at 48 h post induction, whereas the wt and the AP variants were barely detectable even after 72 h of expression (Fig. 2e). These findings demonstrate the intrinsic property of hyperphosphorylated tau to be secreted to the extracellular space from a wide range of experimental models and further supports the suitability of our system for a more detailed analysis of the involved elements.

### Tau is actively secreted via type I UPS mechanisms as a free soluble protein

**Figure 3:**
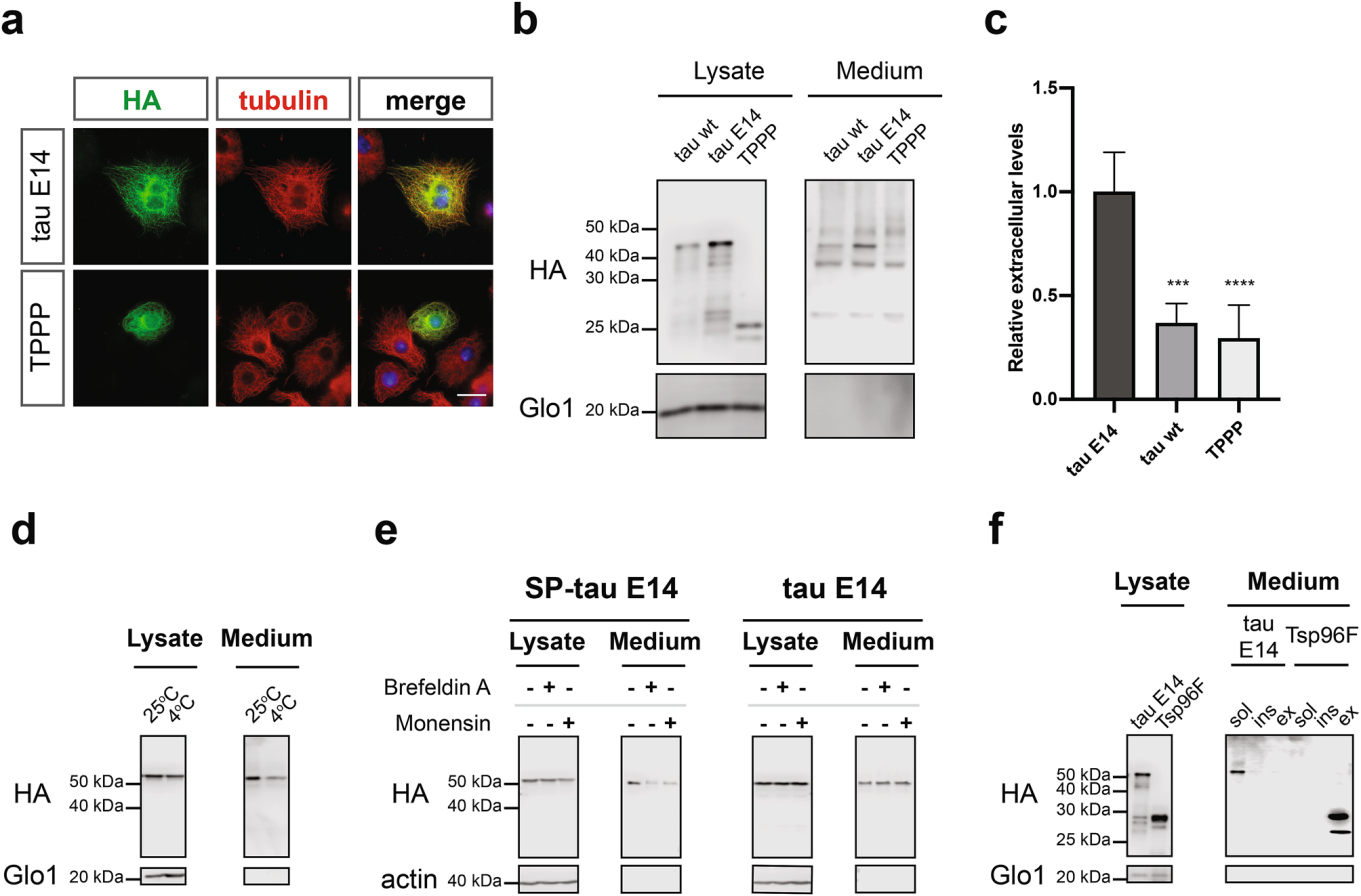
Phosphomimetic tau is secreted as a free protein via UPS Type I mechanisms. **(a)** Cells expressing tau E14 and TPPP were stained using HA antibody, while Hoechst dye was employed for the nucleus (scale bar, 15 µm). **(b,c)** Lysates and immunoprecipitated media were blotted against HA and Glo1. The densitometrically quantified secreted levels were initially normalized to the cell lysates and subsequently compared to tau E14. The data represent mean values ± s.d. derived from at least 3 biological replicates and were subjected to one-way ANOVA, followed by Tukey’s post hoc test. **(d)** Cell lysates and immunoprecipitated media from cells cultured for 6 h at normal conditions (25 °C) or at 4 °C were immunoblotted against HA and Glo1. **(e)** Lysates and immunoprecipitated medium from treated and untreated cells were immunoblotted against HA and actin. **(f)** Sarkosyl-insoluble and exosomal-associated proteins were isolated from conditioned media, while soluble and vesicle-free fractions were subjected to immunoprecipitation. All fractions were immunoblotted against HA, while Glo1 was used as loading and quality control.

To further address the secretion specificity of the phosphomimetic tau E14, we employed as an additional control a different microtubule-associated protein, namely the Tubulin Polymerization-promoting Protein^46^ (TPPP, Fig. 3a). Even though both proteins demonstrated a similar tubulin localization profile upon expression in *Drosophila* neuronal cells (Fig. 3a), TPPP was barely detectable in the conditioned medium (Fig. 3b), with levels similar to these of tau wt and significantly lower than those of tau E14 (Fig. 3c). Moreover, we investigated the dependence of tau secretion on temperature as an additional criterion for active secretion mechanisms. For this, the *Drosophila* neuronal cells expressing the phosphomimetic tau were incubated with fresh medium at 25 °C or 4 °C for 6 h. As expected, we observed a small reduction of the intracellular tau after this short treatment (Fig. 3d). However, this change in culturing conditions led to a significant reduction of the extracellular tau levels by approximately 60% (Fig. 3d and Fig. S2a), suggesting the specificity of tau secretion as an active cellular process.

Subsequently, we questioned the potential contribution of the canonical secretory pathway using two specific inhibitors, namely Brefeldin A and Monensin. The efficacy of those compounds was assessed by treating cells expressing a tau E14 version that was re-directed to the ER/Golgi upon fusing the signal peptide sequence of the *Drosophila* Larval Cuticle Protein 1 (LCP1)^47^ at the N-terminus of tau E14 (SP-tau E14, Fig. S2b). While the treatment with Brefeldin A and Monensin effectively reduced the secretion of the SP-tagged version, none of them had any influence on the cellular localization and secretion of tau E14 (Fig. 3e, Fig. S2b and c). Finally, we examined the contribution of the Type III UPS pathway on the secretion of tau E14 in our newly developed *Drosophila* neuronal culture system, as tau secretion has been associated with such vesicles^23^. The successful isolation of these fractions from the conditioned media was validated through the employment of the exosome-associated Tsp96F protein^48^ (Fig. 3f and Fig. S2d). We were able to detect only minor amounts of insoluble or exosome-associated extracellular tau E14, while the vast majority was retained as free and non-vesicle associated protein (Fig. 3f). Taken together, these data demonstrate that active Type I secretory mechanisms mediate the direct translocation of phosphorylated tau across the plasma membrane as a free and soluble protein.

### Translocation to the extracellular space relies on freely-diffusing cytoplasmic pools of tau

**Figure 4:**
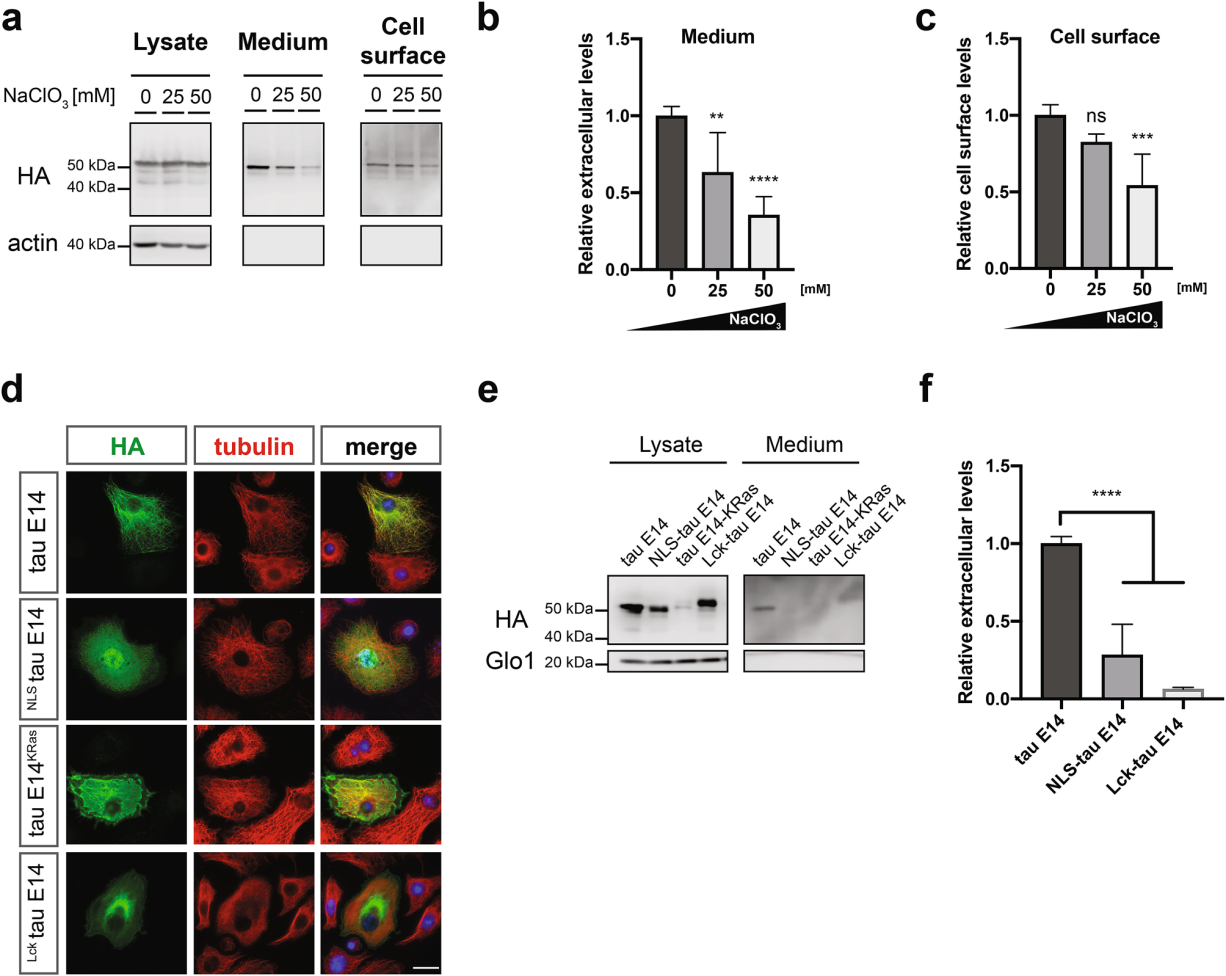
Type I unconventional secretion of tau depends on the presence of functional sulfated proteoglycans. **(a)** Tau E14-expressing cells were treated with increasing concentrations of NaClO_3_ and upon collection of the conditioned media, the living cells were incubated with Heparin to retrieve the surface-bound proteins prior to cell lysis. Both medium and Heparin fractions were subjected to immunoprecipitation and were immunoblotted together with lysates against HA and actin. **(b,c)** Secreted and surface-associated fractions were densitometrically quantified, normalized to the intracellular levels, and then compared to the untreated control. The data represent mean values ± s.d. derived from n = 7 biological replicates for the medium and n = 5 for the Heparin data. Statistical analysis was performed through one-way ANOVA and followed by Tukey’s post hoc test. **(d)** Cells expressing tau E14 with and without cellular localization signals were stained against HA and tubulin, whereas Hoechst was used for nuclear staining (scale bar, 15 µm). **(e,f)** Cell lysates and immunoprecipitated media from cells expressing the tau E14 variants were immunoblotted against HA and Glo1. The densitometrically quantified secreted tau was initially normalized to the intracellular levels and then compared to the original tau E14 variant. The data represent mean values ± s.d. derived from at least 3 biological replicates and were subjected to one-way ANOVA, followed by Tukey’s post hoc test.

We and others have previously demonstrated that the sulfated proteoglycans (PGs) are not only essential for the uptake of tau^49^, but they are also critically involved in the Type I secretion of tau to the exterior^16,17^. To address their contribution on the secretion of tau from *Drosophila* cells, we subjected them to increasing concentrations of sodium chlorate (NaClO_3_), a molecule that blocks the last sulfation step of all PGs^50^. Even though the treatment with NaClO_3_ did not affect the intracellular levels of tau (Fig. 4a), the recovered amount from the conditioned medium demonstrated a dose-dependent reduction, which was already significant at a concentration of 25 mM (Fig. 4a,b). Notably, the surface-retained portion of tau E14, which was retrieved through heparin wash of living cells, was only mildly affected at the same concentration, but displayed a greater decrease (approximately 50%) upon treatment with 50 mM NaClO_3_ (Fig. 4a,c).

Furthermore, the interaction of tau with the inner plasma membrane leaflet has been suggested to represent an important factor for the efficient translocation of the protein to the extracellular space^16,17^. Taking this into consideration, we hypothesized that redirecting tau to distant cellular compartments would inhibit its release from cells, whereas the redirection to the plasma membrane could potentially enhance its secretion. To address this, we generated artificial tau E14 constructs that re-routed the protein to the nucleus or the plasma membrane through nuclear localization (NLS) or plasma membrane targeting signals^51^, respectively (Fig. 4d). The latter was initially attempted through fusing the C-terminal signal of the K-Ras4B^52^ to tau E14, but the chimeric protein was poorly expressed (Fig. 4e), potentially due to stability issues. This problem was bypassed by employing the N-terminal sequence of the tyrosine kinase Lck protein, which drives plasma membrane targeting through lipid modifications, such as palmitoylation, myristoylation and S-acylation^53,54^. As expected, forcing the protein to localize in the nucleus significantly impaired the secretion efficiency of tau E14 (Fig. 4e,f). Intriguingly though, the re-direction of tau to the plasma membrane via the Lck-targeting signal also led to reduced levels of secreted protein (Fig. 4e,f). Taken together these findings indicate that the enforcement of tau to dock at the plasma membrane is not sufficient to promote its secretion to the extracellular space and suggest that the translocation process relies on the diffusion of the tau protein in the cytoplasm to allow the interaction with the PI(4,5)P_2_ at inner leaflet of the plasma membrane.

### The two cysteines of tau are a critical *cis*-element in its unconventional secretion pathway

**Figure 5:**
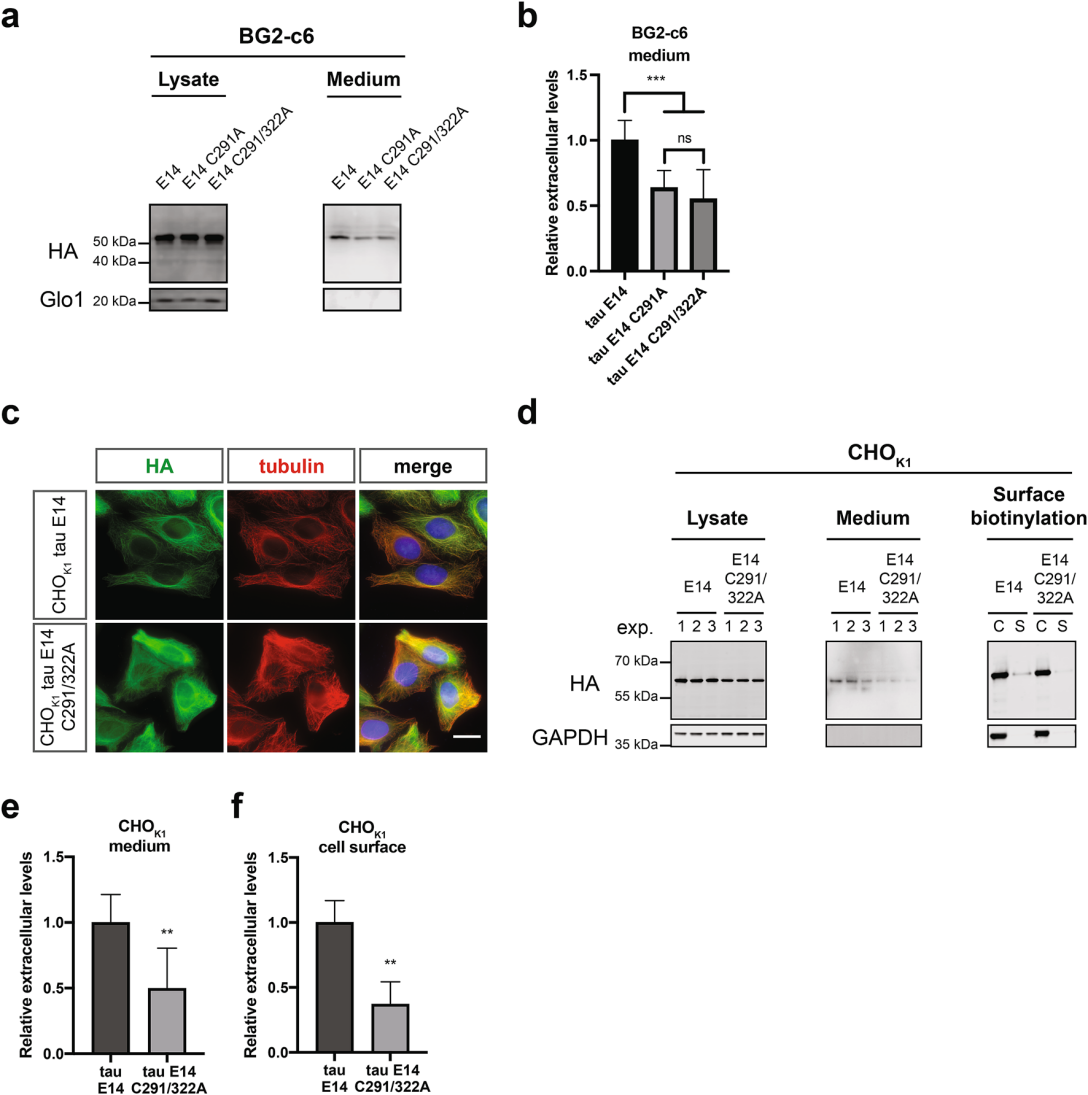
Cysteines in tau are a critical cis-element for its efficient secretion. **(a,b)** Conditioned media from *Drosophila* neuronal cells expressing either tau E14 and the corresponding single or double-cysteine mutant version (C291A and C291/322A, respectively). were immunoprecipitated and together with the cell lysates samples were immunoblotted against HA and Glo1. The secreted amounts were densitometrically quantified, normalized to the intracellular levels and then compared to the tau E14 levels. The data represent mean values ± s.d. derived from n = 10 biological replicates and were subjected to unpaired t test that was followed by Welch’s correction. **(c)** CHO_K1_ cells expressing tau E14 or E14 C291/322A were fixed and stained for tau, tubulin, and nucleus using antibodies against HA, tubulin, and the Hoechst dye respectively (scale bar, 15 µm). **(d)** The conditioned medium from CHO_K1_ cells expressing tau E14 or tau E14 C291/322A was subjected to immunoprecipitation, while cell surface biotinylation was performed on living cells to isolate the cell lysates (C) and the surface-associated (S) fractions. All samples were immunoblotted against HA for the detection of tau, while GAPDH was used as loading, cell integrity, and assay quality control. **(e,f)** The densitometrically quantified secreted amounts in the medium were initially normalized to the expression levels and subsequently compared to tau E14, while the surface-associated fractions were compared to the total expression levels. In both cases, the data represent mean values ± s.d. derived from n = 8 and 4 biological replicates, respectively. Statistical analysis was performed via unpaired t test, which was followed by Welch’s correction.

The interaction of proteins with the plasma membrane is a critical step in the translocation process within the Type I UPS^16,29,55^. The most well-characterized representative is FGF2, where the two surface cysteines (C77 and C95) govern the efficient secretion of the protein through the formation of membrane-inserted intermolecular disulfide bridges^35^. Intriguingly, depending on the resulted isoform due to the alternative splicing of the second repeat, tau may also contain up to two cysteines (C291 and C322, Fig. S2e) that have been associated with the formation of intra- or intermolecular disulfide bonds. To address the impact of these two residues on the secretion propensity of tau we generated two mutant versions of tau E14 by mutating the first cysteine (tau E14 C291A) or both cysteine residues to alanine (tau E14 C291A/322A, Fig. S2e), thereby either allowing exclusively the formation of intermolecular disulfide bridges or completely blocking them, respectively. Even though the expression levels were similar among the different tau E14 variants (Fig. 5a), the secretion efficiency of both the single and the double-cysteine mutant variants was almost half compared to the one with all available cysteines (Fig. 5a,b). Most intriguingly, we observed no significant differences in the secreted levels between the single and the double cysteine tau E14, indicating that the presence of one cysteine and therefore the formation of intermolecular disulphide bridges between tau molecules is not sufficient to mediate tau secretion. Aiming to validate these findings, we generated stable cell lines in our previously established CHO cell culture system^16^. Similar to the results obtained from the *Drosophila* neuronal cells, the cellular localization of the double cysteine-mutant variant was indistinguishable from tau E14 (Fig. 5c) and the expression levels between them were also comparable (Fig. 5d). However, the secreted levels of tau E14 C291/322A in the culture medium were reduced by approximately 50% when compared to that of tau E14 (Fig. 5d,e). In line with the observations in the *Drosophila* neuronal cells, mutation of cysteine-291 to alanine also resulted in a stark reduction of the tau E14 in the culture medium, but without any significant difference compared to the double-cysteine mutant (Fig. S3a and b). Additionally, the surface-retained population of the double-cysteine mutant tau E14 as determined by surface biotinylation experiments on living cells was even further reduced by 65% (Fig. 5d,f). As the sulfated PGs represent an important factor in the secretion of tau (Fig. 4a–c), we analysed whether our newly developed CHO double-cysteine tau E14 cell line has altered levels of sulfated PGs. However, both the levels and the distribution of cell surface-associated sulfated PGs was indistinguishable between the tau E14 and tau E14 C291/322A cells (Fig. S3c). These findings demonstrate that the reduced Type I UPS secretion of tau in the cysteine mutant variants is solely attributed to these residues and underlines the importance of this novel *cis*-element in the translocation process.

### Cell-to-cell spreading of tau relies on the availability of cysteines

**Figure 6:**
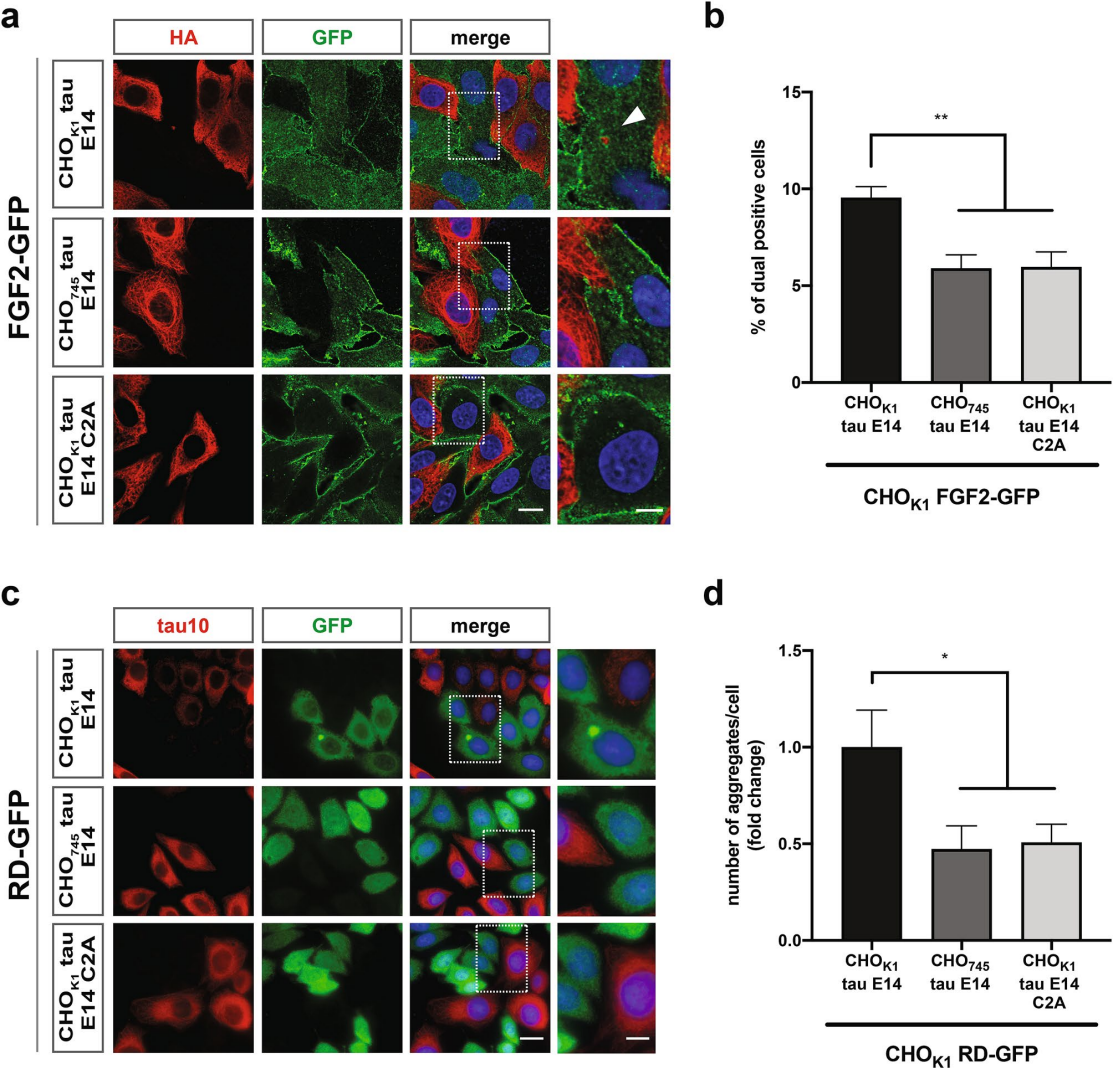
The availability of cysteines drastically influences the trans-propagation of tau. **(a)** CHO_K1_ cells expressing either tau E14 or tau E14 C291/322A were co-cultured with FGF2-GFP cells for 48 h and were subsequently stained for tau, FGF2, and nucleus using antibodies against HA, GFP, and the Hoechst dye, respectively (scale bars, 10 µm and 3 µm for the magnified image). **(b)** The tau-positive signal inside FGF2-GFP cells was quantified through an image-based analysis (3 independent biological replicates with at least 400 processed cells per case). The data represent mean values ± s.d. and were statistically analysed through one-way ANOVA followed by Tukey’s post hoc analysis. **(c)** CHO_K1_ RD-GFP cells were co-cultured with CHO_K1_ tau E14, CHO_745_ tau E14, and CHO_K1_ tau E14 C291/322A cells. All combinations were stained with tau10 and GFP antibodies for full-length tau and RD-GFP detection, respectively. Hoechst dye was used for nuclear staining. Scale bars correspond to 15 µm and 5 µm for the original and magnified images, respectively. **(d)** The RD-GFP positive inclusions were quantified through a semi-automated image analysis of randomly selected z-projected scans and the number of inclusions/cell for each condition was compared to the CHO_K1_ tau E14-CHO_K1_ RD-GFP co-culture combination (3 independent biological replicates with at least 330 processed cells per case). The data represent mean values ± SEM and were statistically analysed through one-way ANOVA followed by Tukey’s post hoc analysis.

The spread of tau pathology relies on the induction of seeded tau aggregation upon trans-cellular propagation in naïve cells^56^. Having demonstrated the significance of the two cysteines of tau in its unconventional secretory mechanism, we tackled the impact of these mutations on the trans-cellular spreading and induction of aggregation in adjacent cells. The first aspect was addressed through co-culturing combinations using our previously established CHO tau cell-to-cell spreading assay^16^. More specifically, in this experimental setup the tau expressing cells (donor line) were co-cultured with cells expressing FGF2-GFP (acceptor line) and we actively searched for tau positive signal inside the acceptor cells as indication of trans-cellular spreading events. In accordance with the secretion phenotypes, the spreading events were easily detectable for tau E14, while the occurrence of tau positive signal inside the acceptor cells was less abundant when the double-cysteine mutant version was employed (Fig. 6a). Levels were quantified through an image-based analysis, where we also employed a control cell line deficient for three out of four sulfated PGs (CHO_745_) and thereby impaired tau trans-cellular spreading efficiency^16,57^. In depth-analysis of these combinations demonstrated that the spreading propensity of the tau E14 C291/322A mutant was almost half of the tau E14 (Fig. 6b), a reduction that is in accordance with the 50% lower extracellular tau levels obtained from these cells.

We next investigated the ability of tau E14 C291/322A to induce aggregation upon trans-cellular spreading to adjacent cells. We employed our CHO-based aggregation biosensor cell line expressing the repeat domain of tau fused with GFP (RD-GFP)^16^. Similar systems have been reported for their high sensitivity in detecting aggregation upon certain stimuli, such as the exogenous administration of recombinant or patient-derived aggregates^14^. Indeed, the liposome-mediated transduction with recombinant filaments efficiently induced the aggregation of the expressed RD-GFP (Fig. S3d and e). In agreement to the cell-to-cell spreading data, the cells expressing the phosphomimetic tau were able to induce the aggregation of RD-GFP in co-culturing conditions (Fig. 6c). However, neither the double-cysteine tau E14 mutant (CHO_K1_ tau E14 C291/322A) nor the sulfated PGs-deficient tau E14 donor cell line (CHO_745_ tau E14) led to such phenotypes (Fig. 6c,d). Taken together, these observations further emphasize the significance of the Type I UPS pathway tau is making use of during trans-cellular propagation and identify the two cysteines residues in tau as a novel *cis*-element required for this pathway.

## Discussion

Over the recent decades the trans-cellular propagation of pathological tau conformations has gained traction as a driver of disease pathogenesis in AD. This has been supported by the increasing evidence associating the elevated levels of tau in the CSF^9^ with the stereotypic deposition of misfolded tau species throughout the brain during disease progression^7^. The proposed mechanisms involve the release of hyperphosphorylated tau species from affected neurons and their uptake from healthy cells, where they seed naïve tau molecules and thereby transmit the aggregated state^11^. The significance of such seeded-templated aggregation events are further highlighted by the structural differences between the endpoint tau amyloid conformations in neurodegenerative diseases, which might in turn define the phenotypic discrepancies between tauopathies^58–60^.

In the current study, we demonstrate a direct link between phosphorylation, de novo aggregation and its associated neurotoxicity in tau pathology. The hyperphosphorylation of tau has been extensively linked to neurodegeneration^6^ and our findings further support this notion, as both the phosphomimetic and the phosphorylated wild-type tau variants induce severe roughness in the fly eye. These toxic phenotypes in the fruit fly have been previously attributed to pathological cell-cycle activation of TOR-mediated pathways^41^ and impaired axonal transport^61^ deriving from soluble cytosolic tau species^43^. However, in accordance with other in vivo systems^62^, our quantitative analysis of the retinal damage at the *Drosophila melanogaster* eye combined with the biochemical characterization of the expressed tau species suggest that the phosphomimetic-induced aggregation of tau E14 substantially contributes to the resulted toxicity. Moreover, our lab as well as others have previously demonstrated that the trans-cellular propagation of misfolded proteinaceous species can be effectively modelled in the fruit fly brain and such events significantly influence the lifespan of these animals^63,64^. As hyperphosphorylation increases the secretion propensity and thereby the cell-to-cell spreading efficiency of tau^16^, we cannot rule out the occurrence of such phenomena that could further enhance the toxicity in the retinal cells should be.

We further addressed the specificity of tau secretion and the mechanistic details governing this process in a *Drosophila* neuronal cell culture system. Although the elevated extracellular CSF-tau levels are often attributed to the passive release of the protein due to neurodegeneration, the endogenous or the ectopic expression of human tau has not been associated to toxic phenotypes in cultured cells^65^. Similarly, in our study we observe similar levels of cell death between control and tau-overexpressing cells. Notably, even though tau E14 was the only tau variant detected in the conditioned medium, there was no apparent difference in toxicity or protein expression levels among the cells expressing the different tau variants, thereby indicating the occurrence of active secretion mechanisms driven by tau phosphorylation. This is further supported by the dependence of tau secretion on temperature changes as a marker of cellular mechanisms mediating the process. On this aspect, we have previously demonstrated that tau is able to interact with components of the plasma membrane in the absence of ATP^16^, a feature also commonly shared among several Type I UPS members^30,55^. Therefore, we envision that while the interaction of tau with lipid components occurs independently of ATP, the steps prior and after these events could energetically rely on cellular functions. However, since the cell line employed in this study is derived from an early developmental stage in the lifespan of the fruit fly^44^ and tau secretion has been shown to rely on neuronal activity in mammalian cultures^66^, a subject of future studies should be to address these phenotypes in differentiated neuronal cells^67^ or in *Drosophila melanogaster*-derived primary neuronal populations^68^.

We have previously shown that the PI(4,5)P_2_ represent one of the lipid components that mediate the translocation process of tau across the plasma membrane^16^. This has been corroborated by further findings linking the secretion of tau with cholesterol and sphingomyelin levels^17^, collectively identifying these three plasma membrane molecules as significant *trans*-elements governing the unconventional secretion of tau. Indeed, when we directed tau away from the plasma membrane to the nuclear compartments, the secretion of the protein was vastly reduced. Surprisingly though, the artificial localization of tau to the plasma membrane through lipid-based targeting motifs^51^ was equally detrimental for the extracellular levels of tau. Interestingly, this is in line with our previous results from the liposome membrane integrity assay, where the replacement of the PI(4,5)P_2_ with Ni–NTA lipids led to a complete abolishment of the membrane activity, despite the recruitment of tau due to its N-terminal His-tag^16^. This suggests that the tau-PI(4,5)P_2_ interaction is required over other forms of membrane recruitment and it further hints towards the existence of dedicated regions within the tau sequence that may be responsible for the efficient translocation. For FGF2 and HIV-Tat the interaction with the PI(4,5)P_2_ is mediated by highly positively-charged residues within their amino acid sequence^29,69^. The tau isoform used throughout this study contains 66 positively charged residues, of which 28 are located within the MTBR. Intriguingly, the disease-associated hyperphosphorylation of tau occurs predominantly in epitopes outside the repeat domain, while leaving these positively charged moieties largely unaffected^16,70,71^. Moreover, a recent study identified the lysine residues within the repeat domains as the primary binding sites to the surface receptor LRP1 *en route* to the internalization of tau from neurons^72^. This suggests that the MTBR of tau can act as an interaction surface for molecules other than the microtubules. Whether this, or other regions of the protein permit the interaction with negatively charged PI(4,5)P_2_ at the inner plasma membrane leaflet remains unknown.

Finally, our work addressed the contribution of the two cysteines (C291 and C322) on the secretion and the trans-cellular spreading of tau. The first cysteine residue is located at the second repeat of the MTBR and therefore it is present only in tau isoforms containing 4 repeat domains, while the second cysteine can be found in all isoforms^36^. These epitopes have been primarily linked to the formation of intra- or inter-molecular disulfide bridges between tau molecules, with the latter being of great interest as a potential initiating step for the formation of tau amyloids^37,73^. Mutation of the first cysteine residue into alanine that allows only the formation of intermolecular disulfide bridges, led to an approximately 50% reduction of secreted tau levels in two independent cell culture systems. Interestingly though, the complete prevention of both intra- and intermolecular disulfide bonds by converting both cysteines into alanine^74^ did not further impair tau secretion. Additionally, in accordance to our previous observations regarding the impact of tau secretion on the trans-cellular propagation^16^, this reduction was also corroborated by decreased cell-to-cell spreading efficiency as well as lower levels of aggregation in our biosensor cell line. We envision that the cysteine-dependent secretion phenotype relies on the formation of intramolecular disulfide bonds, which would in turn provide a tighter folding of the tau molecules into temporary and dynamic structures that allow the completion of the translocation process^75,76^. Notably, this mechanism would distinguish tau from the pathway by which FGF2 is secreted^24^, where the cysteine-dependent oligomerization is a critical determinant that drives the translocation process^35^. However, as the secretion potency of the cysteine mutant versions of tau was not fully depleted, we cannot rule out the contribution of other *cis*-elements on the tau sequence mediating this process and could potentially foster the formation of cysteine-independent oligomeric assemblies^74^ that are able to translocate to the extracellular space. Furthermore, a limitation of our studies is the employment of a single tau isoform and future work should systematically determine the contribution of the second repeat domain in the MTBR as well as the N-terminal projection domains, especially since the latter have been directly associated to interact with the plasma membrane^77^.

Collectively, the herein described findings further advance our understanding on the mechanistic details governing the Type I unconventional secretion of tau. We propose that specific residues within the tau sequence mediate the interaction with the inner plasma membrane leaflet. Notably, we demonstrate that the two cysteine residues of full-length tau act as key mediators of this phenotype, thereby identifying a novel *cis*-element governing the unconventional secretion of tau. Therefore, we suggest that the disease-associated redox disturbances in physiological cellular homeostasis might not only be a source of neuronal damage^78^, but they could in turn drive and promote the trans-cellular propagation of tau. Most importantly, such efforts for the in-depth characterization of the molecular mechanisms involved in the initiation and progression of the disease-related pathology will provide a stepping stone in the design of therapeutic approaches against neurodegeneration.

## Methods

All chemicals and culture media were purchased by Sigma-Aldrich, unless otherwise mentioned. The statistical analyses shown in the study were performed using the GraphPad Prism software (GraphPad Software) with the following significances: ^ns^p > 0.05; ^*^p ≤ 0.05; ^**^p ≤ 0.01; ^***^p ≤ 0.001; and ^****^p ≤ 0.0001.

### *Drosophila melanogaster* transgenic lines and husbandry

All flies were raised on standard cornmeal and molasses medium. Crosses were kept for three days at 25 °C before shifting them to 29 °C. The progenies were collected within a window of 24 h and mated female flies were kept at 29 °C. All flies were age matched when used for experiments. The expression of all constructs at the retinal cells was achieved using the X-GMR-Gal4 driver line, which we acquired from the Bloomington *Drosophila* Stock Center (FlyBase ID: FBti0072862). The tau lines were kindly provided by the M. Feany lab (UAS-tau wt^38^ FlyBase ID: FBtp0014839, UAS-tau E14^41^ FlyBase ID: FBtp0055829, UAS-tau AP^40^ FlyBase ID: FBtp0055953). The UAS-eGFP line was acquired from the Bloomington *Drosophila* Stock Center (FlyBase ID: FBti0013988).

### Toxicity assay at the *Drosophila* eyes

The *Drosophila melanogaster* flies expressing the tau variants or GFP were moved to 29 °C after eclosion and analysed at 2 or 3 days of age. For image acquisition, the flies were initially anesthetized with CO_2_ and gently laid on a drop of nail polish to immobilize them. The *Drosophila* heads were photographed using a Zeiss STEMI 2000-C binocular epifluorescence microscope and Axiocam camera. The severity of the roughness at the fly eye was quantified using the ImageJ open-source plugin *Flynotyper*^79^ and all values were finally compared to the GFP control line.

### Sarkosyl-insoluble proteins isolation

For the in vivo sarkosyl-insoluble fractions, 6 mated flies per genotype were fresh-frozen in liquid nitrogen and decapitated. The frozen heads were homogenized in 6.7 µl/head ice-cold Buffer A (50 mM Tris–HCl pH 7.5, 2 mM Na_3_VO_4_, 50 mM NaF, 50 mM β-Glycerophosphate, 150 mM NaCl, 2 mM MgCl_2_, 1 × cOmplete EDTA-Free protease inhibitors mix, 1 × PhosSTOP phosphatase inhibitors mix) for 45 s on maximum speed using a Minilys homogenizer (Bertin Instruments). The homogenate was centrifuged at 6000*g* for 5 min at 4 °C and 34 µl of clarified homogenate was isolated. The homogenization process was repeated using an equal amount of Buffer A and 34 µl of clarified homogenate were combined with the first round, while the pellet was discarded. Subsequently N-lauroylsarcosine, Triton X-100, and SDS were added to final concentrations of 1% w/v, 1% w/v, and 0.1% w/v, respectively (Sarkosyl-Lysis Buffer). Upon the final addition of β-MeOH to 0.1 M final concentration (1% v/v), the samples were shortly vortexed and incubated at 37 °C for 1 h under 700 rpm orbital shaking. Then, the samples were centrifuged at 4 °C for 30 min at 21,000*g*. The supernatant corresponding to the soluble proteins was isolated and mixed with Lämmli, while the pellet was resuspended with an equal volume of Buffer A and the centrifugation step was repeated. This wash-supernatant was discarded and the pellet was resuspended in 50 µl Lämmli. Both fractions were boiled for 10 min at 95 °C and analysed in SDS-PAGE and western blotting.

The Drosophila neuronal BG2-c6 cells were cultured in 24-well plates for 48 or 72 h upon Cu^2+^ induction and were initially rinsed with PBS and before gently detached by resuspension in PBS. The cells were shortly pelleted (5000*g*, 5 min, 4 °C) and then lysed for 30 min on ice with Sarkosyl-Lysis Buffer. The lysate was centrifuged at 21,000*g* for 30 min at 4 °C and the supernatant (soluble fraction) was separated and mixed with Lämmli. The pellet was washed with 50 µl Sarkosyl-Lysis Buffer, centrifuged again and resuspended in Lämmli. Both soluble and insoluble fractions were boiled at 95 °C for 10 min before SDS-PAGE and western blotting analysis.

### Genomic DNA isolation from tau transgenic *Drosophila melanogaster* flies

Two flies from each genotype were shortly anesthetized with CO_2_ and transferred into a tube on ice. The flies were squished using a pipette tip in 50 μl of Genomic DNA Extraction Buffer (10 mM Tris–HCl pH  8, 1 mM EDTA, 25 mM NaCl, supplemented immediately before use with 200 μg/ml Proteinase K). The reaction was incubated for 30 min at 37 °C and subsequently boiled at 95 °C for 2 min to inactivate the Proteinase K. From this mixture 1–2 μl were used in a PCR for amplifying the tau coding sequences.

### Cell lines maintenance

*Drosophila melanogaster* neuronal BG2-c6 cells (ML-DmBG2-c6) were obtained from the Drosophila Genomics Resource Center (DGRC) and were maintained in Schneider’s Drosophila medium (Thermo Fisher Scientific), supplemented with 10% FBS, 100 IU/ml penicillin, and 100 µg/ml streptomycin, and 10 µg/ml insulin at 25 °C, and atmospheric conditions. Chinese Hamster Ovarian (CHO) cells were maintained in α-modified Minimum Essential Medium (MEM-a), supplemented with 10% fetal calf serum (FCS, Biochrom AG), 2 mM glutamine, 100 IU/ml penicillin, and 100 µg/ml streptomycin at 37 °C, 5% CO_2_ and 95% humidity. Human Embryonic Kidney (HEK) EcoPack 2–293 cells were maintained in collagen-coated (Collagen R, Serva Electrophoresis) plates with Dulbecco’s modified Eagle Medium (DMEM, Sigma-Aldrich), supplemented with 10% FCS, 100 IU/ml penicillin, and 100 µg/ml streptomycin at 37 °C, 5% CO_2_ and 95% humidity. Sf9 cells were grown in roller culture bottles in Sf-900 II serum-free medium (Thermo Fisher Scientific) at 27 °C under shaking at atmospheric conditions.

### Stable cell lines generation

CHO_K1_ cells stably expressing the tau E14 C291A and tau E14 C291/322A variants were generated as described previously^16^. Briefly, as target cells the genetically modified CHO_K1_ cell line was employed^80^, which expresses the murine cationic amino acid transporter MCAT-1^81^ and the doxycycline-sensitive transactivator rtTA2-M2^82^. For retrovirus production, the MBS Mammalian Transfection Kit (Agilent Technologies) was used on EcoPack 2–293 cells according to manufacturer’s instructions. The day before transfection, the cells were seeded in collagen-coated 10 cm dishes in such density that the next day they would reach 80% confluency. The cells were transfected with 9 µg retroviral plasmid containing the gene of interest and after production of the viruses, they were delivered to the CHO_K1_ target cells according to manufacturer’s instructions, adapting the recommended volumes as needed. Upon viral transduction, the target cells were selected by 400 µg/ml Hygromycin B for approximately 2–3 weeks. The CHO_K1_ FGF2 wt-GFP, CHO_K1_ tau E14, CHO_745_ tau E14, and the CHO_K1_ RD-GFP cell lines were previously generated^16,32^. Expression of the desired proteins in the stable CHO cells was induced by addition of doxycycline into the medium to a final concentration of 1 µg/ml.

### Plasmids and primers

The transient transfections of the BG2-c6 cells were performed using the pMT-puro vector (Addgene plasmid #17923). The coding sequences of the tau variants were subcloned using the following pair of primers: tau_KpnI_fwd (TAGCTGGTACCTACCAATCAAATGGCTGAGCCCCGCCAGGAG) and tau_3xHA_NotI_rev (CTGAGCGGCCGCTTATTAAGCGTAATCTGGAACATCGTATGGGTAAGCGTAATCTGGAACATCGTATGGGTAAGCGTAATCTGGAACATCGTATGGGTACAAACCCTGCTTGGCCAGGG). The single-cysteine mutant version of the tau E14 variant (tau E14 C291A) was generated by replacing the cysteine residue at position 291 and the double-cysteine variant (tau E14 C291/322A) by converting both epitopes at positions 291 and 322 (numbering is based on the longest tau 2N4R isoform) into alanines through site-directed mutagenesis using the following pairs of primers: tau C291A_sense (tagcaacgtccagtccaaggctggctcaaaggataatatc), tau C291A_antisense (gatattatcctttgagccagccttggactggacgttgcta), tau C322A_sense (gcaaggtgacctccaaggctggctcattaggcaaca), and tau C322A_antisense (tgttgcctaatgagccagccttggaggtcaccttgc). The tau E14 cysteine mutant variants were then subcloned into the pMT-puro vector using the aforementioned tau_KpnI_fwd and tau_3xHA_NotI_rev primers, while for the stable cell lines generation, the coding sequences were subcloned into the pRTi-TRE2 vector^16^ with the following pair of primers: tau_pRTi_BamHI_fw (TAGCTGGATCCTACGCCACCATGGCTGAGCCCCGCCAGGAG) and tau 3xHA_pRTi_PacI_rev (CTGATTAATTAATTATTAAGCGTAATCTGGAACATCGTATGGGTAAGCGTAATCTGGAACATCGTATGGGTAAGCGTAATCTGGAACATCGTATGGGTACAAACCCTGCTTGGCCAGGG). The pCMV6-myc-TPPP plasmid (Origene plasmid #RC213142) was used to amplify the coding sequence of TPPP, which was subcloned to the pMT-puro vector using the following set of primers: TPPP_KpnI_fw (TAGCTGGTACCTACCAATCAAATGGCCGATAAGGCCAAGC) and TPPP_3xHA_NotI_end_rev (CTGAGCGGCCGCTTATTAAGCGTAATCTGGAACATCGTATGGGTAAGCGTAATCTGGAACATCGTATGGGTAAGCGTAATCTGGAACATCGTATGGGTACTTGCCGCCCTGCACC). The pMT-HA-Tsp96F was a kind gift from Michael Boutros^48^ and was used to amplify the coding sequence of Tsp96F with the following pair of primers: Tsp97_KpnI_fw (TAGCTGGTACCTACCAATCAAATGggtctcaacggctgctgttcg) and Tsp97_3xHA_NotI_rev (CTGAGCGGCCGCTTATTAAGCGTAATCTGGAACATCGTATGGGTAAGCGTAATCTGGAACATCGTATGGGTAAGCGTAATCTGGAACATCGTATGGGTAggccttgtagtgctgattcct). The cellular localization signals to generate the chimeric tau E14 variants were introduced by amplifying the tau E14 cDNA with the aforementioned tau_3xHA_NotI_rev primer and the following forward primers: tau_LCP1_SP_fw (TAGCTGGTACCTACCAATCAAATGTTCAAGTTCGTGATGATCTGCGCCGTGCTGGGCCTGGCCGTGGCCGCTGAGCCCCGCCAGGAG), tau_NLS_KpnI_fwd (TAGCTGGTACCCAATCAAATGCGCAGCCGCGCCGATCCCAAGAAGAAGCGCAAGGTGGATCCCAAGAAGAAGCGCAAGGTGGAGCCCAAGAAGAAGCGCAAGGCTGAGCCCCGCCAGGAG), and tau_Lck_KpnI_fwd (TAGCTGGTACCTACCAATCAAATGGGCTGCGGCTGCAGCAGCCACCCCGAGGCTGAGCCCCGCCAGGAG). The tau E14 kRas construct was generated by using the following set of primers: tau_3xHA_KpnI_fwd (TAGCTGGTACCTACCAATCAAATGTACCCATACGATGTTCCAGATTACGCTTACCCATACGATGTTCCAGATTACGCTTACCCATACGATGTTCCAGATTACGCTGCTGAGCCCCGCCAG) and tau_K-Ras4B_NotI_rev (CTGAGCGGCCGCTTATTACATCACGGTGCAGCGGGTGCGGCTCTTCTTCTTCTTCTTCTTGCCATCCTTGCTCAAACCCTGCTTGGCCAGGG).

### Transient transfections

The BG2-c6 cells were transiently transfected using the Effectene Transfection Reagent (Qiagen) according to the manufacturer’s instructions. Briefly, confluent cells were detached by gentle tapping and resuspension. For one well of a 24-well plate, 100 ng of plasmid were diluted in Buffer EC and 1.6 µl of the Enhancer was added. The mixture was vortexed and incubated at RT for 5 min. Subsequently, 5 µl of the Effectene Transfection Reagent was added and the solution was vortexed for 10 s. The reaction was incubated for 10 min at RT and complete medium was added up to 350 µl. The complexes were mixed with an equal volume of cells at a final concentration of 1.5 10^6^ cells/ml and they were plated on concanavalin A (ConA, 0.5 mg/ml)-coated plates. After 6 h, the complexes were removed and replaced with fresh medium containing 1 mM CuSO_4_ for expression induction (unless indicated otherwise for titration experiments). For immunofluorescence (IF) experiments the transfected cells were plated on ConA-coated glass cover slips and transfected following the same procedure.

### Antibodies

**Table:** 

Antibody	Source	WB	IF
Rabbit polyclonal anti-HA	Sigma-Aldrich	1:1000	1:500
Mouse monoclonal alpha-tubulin	Developmental Studies Hybridoma Bank	–	1:200
Rabbit polyclonal anti-actin	Sigma-Aldrich	1:3000	–
Rabbit polyclonal anti-Glyoxalase 1	Santa Cruz Biotechnology	1:3000	–
Chicken polyclonal anti-GFP	Aves Labs Inc	1:1000	1:300
Mouse monoclonal tau A-10	Santa Cruz Biotechnology	–	1:200
Mouse monoclonal anti-HSPGs 10E4	Biomol		1:100
Mouse monoclonal anti-GAPDH	Thermo Fisher Scientific	1:15000	–
Rabbit polyclonal anti-tau KJ9A	Agilent Technologies/Dako	1:5000	–
Alexa Fluor goat anti-Rabbit 488	Thermo Fisher Scientific	–	1:1000
Alexa Fluor goat anti-Rabbit 546	Thermo Fisher Scientific	–	1:1000
Alexa Fluor goat anti-Mouse 546	Thermo Fisher Scientific	–	1:1000
Alexa Fluor goat anti-Chicken 488	Thermo Fisher Scientific	–	1:1000
Alexa Fluor goat anti-Mouse 680	Thermo Fisher Scientific	1:5000	–
IRDye 800CW goat anti-Rabbit	LI-COR Biosciences	1:5000	–
HRP-conjugated goat anti-Rabbit	Thermo Fisher Scientific	1:2000	–
HRP-conjugated goat anti-Mouse	Thermo Fisher Scientific	1:2000	–
HRP-conjugated goat anti-Chicken	Thermo Fisher Scientific	1:2000	–

### In vitro dephosphorylation

BG2-c6 cells were transfected and after the indicated expression time points were rinsed once with PBS and then detached by gentle resuspension with PBS. The cells from 2 wells were mixed and divided in two equal parts before pelleting (1000*g*, 10 min, 4 °C). Subsequently, they were lysed with either PPase-lysis buffer (25 mM HEPES pH 7.4, 300 mM NaCl, 10% Glycerol, 1 × EDTA-Free Protease Inhibitor mix, 1% w/v NP-40) or Lysis Buffer (25 mM HEPES pH 7.4, 300 mM NaCl, 10% Glycerol, 10 mM EGTA, 50 mM NaF, 2 mM Na_3_VO_4_, 50 mM β-Glycerophosphate, 1 × EDTA-Free Protease Inhibitor mix, 1 × Phosphatase Inhibitor mix, 1% w/v NP-40) and incubated at 4 °C for 30 min. The lysates were clarified by centrifugation (14,000*g*, 15 min, 4 °C) and incubated with Antarctic Phosphatase for 1 h at 37 °C. The reactions were stopped by the addition of Lämmli buffer and boiling at 95 °C for 10 min.

### Isolation of secreted proteins in the medium

The conditioned medium was clarified through a series of centrifugation steps (all at 4 °C) to isolate the free proteins^83^: 300*g*, 10 min with the pellet corresponding to living floating cells; 2000*g*, 10 min with the pellet corresponding to dead cells; 10,000*g*, 30 min with the pellet corresponding to cell debris; 21,000*g* 30 min with the pellet corresponding to insoluble proteins. For exosomes isolation, the medium after the last clarification step was subjected to ultracentrifugation (100,000*g*, 70 min, 4 °C). Both insoluble and exosomal fractions were washed once with PBS and re-pelleted with the same conditions before resuspension in Lämmli and boiling for 10 min at 95 °C. Equal volumes of the supernatant fractions corresponding to the clarified medium were subjected to immunoprecipitation using the KJ9A pan-tau antibody.

The day before harvesting the medium, the pan-tau antibody was coupled to Dynabeads Protein G. For this purpose, 30 µl of properly mixed beads for each reaction were mixed with 5 µg of polyclonal anti-tau antibody diluted in 200 µl PBS-T. The antibody-beads mixture was incubated with rotation overnight at 4 °C. The next day, the Dynabeads were magnetically separated from the solution and the supernatant was discarded. The antibody-coupled beads were initially washed twice with 200 µl per reaction of PBS-T and then twice with 250 µl of Conjugation Buffer (20 mM Na_2_HPO_4_, 150 mM NaCl, pH 7–9). The equilibrated antibody-coupled beads were incubated while rotating for 2 h at RT with freshly prepared 5 mM of BS^3^ (bis(sulfosuccinimidyl)suberate) cross-linker, diluted in Conjugation Buffer. At the end of the incubation, the reaction was quenched by addition of 12.5 µl Quenching Buffer (1 M Tris–HCl pH 7.5) per reaction and incubated for 30 min with rotation. The beads were subsequently washed three times with 200 µl PBS-T per reaction and finally resuspended with the clarified conditioned medium. The immunoprecipitation was performed overnight at 4 °C with constant rotation. The next day, the beads were washed three times with 200 µl PBS-T and transferred to a new tube before the elution. The immunoprecipitated proteins were eluted by addition of 30 µl of IP Elution Buffer (25 mM Citrate pH 2.4) and incubation for 10 min at RT while rotating. At the eluted fraction 7.5 µl of IP Neutralization Buffer (0.5 M Tris–HCl pH 8.3) and 7.5 µl of 4 × Lämmli sample buffer were added (final volume 45 µl) and the mixture was boiled for 10 min at 95 °C. After subjecting the secreted tau to western blotting, they were densitometrically quantified and normalized to the intracellular expression levels, which was defined as the ratio between tau and loading control (actin or Glo1 for BG2-c6 and GAPDH for CHO_K1_ cells) in the lysate fractions. Finally, the normalized secreted tau levels were compared to the corresponding reference condition for each experiment and plotted accordingly for statistical analysis.

### Cell-surface biotinylation

Approximately 250,000 CHO_K1_ cells (0.8 × 10^5^ cells/ml) were seeded in 6-well plates and induced with 1 µg/ml doxycycline. After 48 h of expression, the cells were transferred on ice, the medium was quickly removed and the cells were rinsed twice with 1 ml PBS Ca/Mg (PBS, 1 mM MgCl_2_, 0.1 mM CaCl_2_). Afterwards they were incubated with 600 µl of 1 mg/ml EZ-Link Sulfo-NHS-SS-Biotin diluted in Biotinylation Incubation Buffer (150 mM NaCl, 10 mM Triethanolamin, 2 mM CaCl_2_, final pH ≥ 9.0) for 30 min on ice while mildly shaking. At the end of the incubation the biotin solution was removed, the cells were rinsed once with 500 µl Biotinylation Quenching Buffer (PBS Ca/Mg, 100 mM Glycine) and then incubated with 600 µl Biotinylation Quenching Buffer on ice for 20 min with mild shaking. Then, the cells were rinsed twice with PBS and lysed with 300 µl Biotinylation Lysis Buffer (50 mM Tris–HCl pH 7.5, 62.5 mM EDTA pH 8.0, 0.4% w/v Deoxycholate, 1% w/v NP-40, EDTA-Free Protease Inhibitor mix) for 10 min at 37 °C. The cells were scraped and the lysate was transferred to a tube. After a 3 min sonication step, the lysate was incubated at RT for 15 min with vortexing every 5 min to solubilize all proteins. This was followed by a centrifugation step at 18,000 g for 10 min at 4 °C and the pellet was discarded. 15 µl from the clarified lysate were mixed with 15 µl of 4 × Lämmli (input of total cell lysate) and boiled for 10 min at 95 °C. For every reaction 40 µl suspension (or 20 µl packed) Streptavidin UltraLink Resin beads were employed, which were pelleted by centrifugation at 3,000 g after every washing and incubation step. The beads were washed 3 times with 300 µl of Biotinylation Lysis Buffer and then incubated for 1 h at RT with the clarified cell lysate. Afterwards they were washed once with 500 µl of Biotinylation Washing Buffer 1 (50 mM Tris–HCl pH 7.5, 62.5 mM EDTA pH 8.0, 500 mM NaCl, 0.4% w/v Deoxycholate, 1% w/v NP-40, 1 × EDTA-Free Protease Inhibitor mix) and three times with 500 µl of Biotinylation Washing Buffer 2 (50 mM Tris–HCl pH 7.5, 62.5 mM EDTA pH 8.0, 500 mM NaCl, 0.4% w/v Deoxycholate, 0.1% w/v NP-40, 1 × EDTA-Free Protease Inhibitor mix). The washed beads at the end were eluted by adding 40 µl 4 × Lämmli buffer and boiling for 10 min at 95 °C (surface fraction). For the SDS-PAGE analysis, 1.6% of the total cell lysate (10 µl) and 30% of the surface biotinylated fraction (20 µl) were loaded. At the downstream analysis, all samples with biotinylated GAPDH more than 0.5% were excluded.

### Treatment of cells with inhibitors

After 66 h of expression the medium was removed, the cells were rinsed once with PBS and new medium was added containing 1 mM CuSO_4_ and 33 µM Brefeldin A or 50 µM Monensin. The cells were incubated for 6 h in the presence of the inhibitors and then the medium was collected, clarified and subjected to immunoprecipitation, while the cells were lysed in Lysis Buffer. The lysate was clarified (14,000*g*, 15 min, 4 °C) and mixed with Lämmli buffer, followed by a boiling step at 95 °C.

For the NaClO_3_ treatment, a pre-treatment step before induction of expression was performed to ensure low sulfated proteoglycans levels at the cell surface throughout the experiment. BG2-c6 cells were transfected as described above and after 6 h the complexes were removed and replaced with fresh medium containing the indicated concentrations of NaClO_3_. After 2 days, the cells were detached by gently tapping and resuspension, counted, and seeded in new ConA-coated plates with 1 mM CuSO4 and the corresponding NaClO_3_ for further 72 h. Afterwards the medium was collected and the surface-retained tau population was retrieved by Heparin-wash. Both fractions were clarified and subjected to immunoprecipitation, while the cells were lysed with Lysis Buffer.

### Purification of recombinant tau E14

The recombinant 6xHis-tau E14 was expressed and purified from Sf9 insect cells using the Baculovirus expression system as previously described^16,84^. Briefly, the cells from two 500 ml cultures were combined, pelleted, and lysed with Purification Lysis Buffer (Lysis Buffer plus 1 mM Benzamidine, 1 mM PMSF, 14 mM 2-mercaptoethanol) by vigorous pipetting, followed by snap-freezing in liquid nitrogen. The frozen lysate was quickly thawed, sonicated using a Sonifier cell disruptor B-30 (Branson Sonic Power) and boiled for 10 min at 95 °C. The lysates were centrifuged (100,000*g*, 1 h, 4 °C) and the supernatant was loaded on a Ni^2+^ affinity column (HisTrap FF, GE Healthcare Life Sciences), The purified protein was concentrated using 10 kDa cutoff Amicon Ultra-4 concentrators and loaded on a Superdex 200 10/300 GL (GE Healthcare Life Sciences) size exclusion chromatography column with HK Buffer (25 mM HEPES–KOH pH 7.4, 100 mM KCl, freshly supplemented with 1 mM DTT) as buffer exchange. The fractions were analysed for purity and quality through SDS-PAGE and total protein staining with Coomassie InstantBlue. The affinity purification and size exclusion chromatography steps were performed using the ÄKTA system.

### In vitro recombinant tau aggregation

Recombinant tau E14 was mixed with freshly prepared Heparin in final concentrations of 15 µM and 3.75 µM (tau:Heparin 4:1). The protein was left to aggregated at 37 °C under constant shaking for 72 h in the presence of Protease inhibitors and 2 mM DTT. The generation of recombinant filaments at the end of the incubation was validated by SDS-PAGE and Thioflavin T analysis. The tau E14 fibrils were sonicated in water bath sonicator for 15 s, aliquoted and snap-frozen before storage at − 80 °C.

### Transduction of CHO RD-GFP cells with recombinant fibrils

The liposome-mediated transduction was conducted as described previously^16^. Approximately 15,000 cells CHO_K1_ RD-GFP cells were seeded in 8-well Lab-Tek chambers (Thermo Fisher Scientific) in the presence of 1 µg/ml doxycycline. The next day, for transducing 2 wells of an 8-well, 24 µl of Opti-MEM I Reduced Serum (Thermo Fisher Scientific) medium was supplemented with 1 µl of Lipofectamine-2000 and these were mixed with 25 µl Opti-MEM I Reduced Serum medium containing the sonicated tau fibrils. As control, monomeric tau E14 was employed. The protein-liposomes mix was incubated at RT for 20 min and then 20 µl for every well were applied drop-wise to the cells. The final concentration of tau fibrils or monomer applied to the cells was 400 nM. About 16 h later, the cells were fixed and processed for analysis.

### Cytotoxicity determination by measuring the LDH activity

The cytotoxicity assessment was performed using the CytoTox 96 Non-Radioactive Cytotoxicity Assay (Promega) according to the manufacturer’s instructions with slight modifications. After transfection with the tau variants and the indicated treatments, the medium was collected and clarified. The cells were rinsed with PBS and then lysed in 180 µl of LDH Lysis Buffer (full Schneider’s medium supplemented with 1 × Lysis Solution, approximately 0.8% Triton X-100 final concentration) for 45 min at RT. In order to measure the maximum LDH activity, 10% from every lysate was used. Full Schneider’s medium was employed as negative control. In a 96-well flat clear bottom plate, 50 µl from every sample was pipetted and mixed with 50 µl of CytoTox 96 Reagent. Every sample was measured in technical triplicates. The reaction was incubated at RT for 30 min protected from light. At the end of the incubation, 50 µl of Stop Solution was added to each well and the absorbance was measured using a FLUOstar Omega plate reader with the wavelength set at 490 nm. The measurement from every sample was corrected by subtracting the background. The cell death levels were calculated as % of the maximum LDH activity derived from the lysed cells.

### Heparin wash of cell surface bound proteins

After 72 h of expression the medium was collected, clarified and used for further analysis. The cells were rinsed once with PBS and then incubated for 10 min on ice mildly shaking with freshly prepared 500 µg/ml Heparin diluted in Schneider’s medium without FBS. The Heparin-retrieved fractions were subjected to immunoprecipitation and analysed as described above.

### Immunofluorescence

For immunofluorescence experiments the transfected BG2-c6 cells were cultured on Con-A coated glass cover slips for 72 h and the CHO cells on 8-well Lab-Tek chambers (Thermo Fisher Scientific) for 48 h. After these time points for both cases, the medium was removed, the cells were rinsed once with PBS and then fixed with ice-cold methanol for 10 min. Afterwards the cells were washed 3 times with PBS and blocked with IF-blocking buffer (5% FCS diluted in PBS) for 30 min. The cells were incubated with appropriate concentrations of primary and secondary antibodies, diluted in IF-blocking buffer, for 1 h respectively, followed by 3 times PBS wash steps for both cases. At the end of the secondary antibody incubation, the cells were stained for 10 min with 1 µg/ml Hoechst 33342. Images were taken with a 63 × objective using a Zeiss Cell Observer widefield microscope and a Zeiss LSM 880 confocal microscope.

The tau trans-cellular spreading and the induction of aggregation analyses were performed as described before^16^. Briefly, for the tau trafficking image analysis the co-culturing combinations were fixed and stained as described above. The imaging acquisition was performed for 3 independent experimental sets and for each experiment 20 randomly selected fields were imaged. The areas were acquired as z-stacks from the top to the bottom of the cells and the image analysis was performed using the Fiji software with a macro developed at the DKFZ Light Microscopy Core Facility (Heidelberg, Germany). After maximal z-projection, the stacks were locally subtracted for background using the Rolling ball algorithm subtraction and maximum intensity was further used for cells segmentation and analysis. The cells were segmented based on nuclear and cytoplasmic staining using the Median filter and Find Maxima tools, with Segmented Particles above lower threshold option activated. Initially the images of nuclei alone were segmented using the same ImageJ tools and excluded from further analysis. The same was applied for the tau expressing cells. Finally, the tau positive signal localized in the cytoplasm of the FGF2-GFP positive cells was counted for each cell cytoplasm separately using the Analyze Particles tool. A minimum of 400 cells per condition was analyzed. Statistical analysis was performed by calculating the % of FGF2-GFP cells with tau signal in their cytoplasm.

For the biosensor seeding image analysis, the transduced RD-GFP cells or the different culture combinations were fixed with 4% PFA (Electron Microscopy Sciences) diluted in PBS for 10 min at RT in order to preserve the GFP fluorescence of the aggregates. After a 10 min permeabilization step with 0.1% w/v Triton X-100, the cells were stained as described above. The imaging acquisition was performed with at least 3 independent experimental sets of single or co-culture combinations and for each experiment 20 randomly selected fields were imaged. The areas were acquired as z-stacks from the top to the bottom of the cells and the image analysis was performed using the Fiji software with a macro developed at the DKFZ Light Microscopy Core Facility (Heidelberg, Germany). The z-stacks were z-projected and locally background subtracted using the Rolling ball algorithm. Maximum intensity was further used for cell segmentation and analysis. Based on nuclear and cytoplasmic staining the cells were segmented using the Median filter and Find Maxima tools, with activated the option of Segmented Particles above lower threshold. The images of nuclei were segmented using the same ImageJ tools and excluded from further analysis. The same was also applied for the tau expressing cells. Finally, in the remaining GFP-positive cells the RD-GFP aggregates were counted for each cell separately using the Analyze Particles tool. A minimum of 1000 cells per condition was analyzed. Statistical analysis was performed by calculating the % of RD-GFP aggregates containing cells.

### SDS-PAGE analysis and western blotting

The protein samples mixed with Lämmli buffer (40% v/v Glycerol, 240 mM Tris–HCl pH 6.8, 8% w/v SDS, 0.04% Bromophenol Blue, 5% v/v 2-mercaptoethanol) were boiled at 95 °C for 10 min before loading. For regular SDS-PAGE analysis, 4–12% NuPAGE gradient gels (Thermo Fisher Scientific) were employed and run in NuPAGE MOPS SDS Running Buffer (Thermo Fisher Scientific) for 55 min at 200 V. For the dephosphorylation and the MassSpec experiments, self-casted 10% or 15% acrylamide gels were used in order to obtain maximum resolution of the differentially phosphorylated tau bands. These gels were run in Towbin SDS-PAGE Running Buffer (25 mM Tris, 192 mM Glycine, 0.1% w/v SDS) until the dye front reached the end of the gel. The analyzed proteins were transferred to Immobilon FL PVDF membranes (Millipore Corporation) and incubated for 1 h at RT with blocking buffer (5% w/v milk in PBS) under shaking. The membranes were washed 3 times with PBS-T and incubated overnight at 4 °C with the primary antibody diluted in Blot Incubation Buffer (1% w/v BSA diluted in PBS-T). The next day, the membranes were washed 3 times for 10 min with PBS-T and incubated for 1 h at RT with the secondary antibody diluted in Blot Incubation Buffer. All membranes (except the insoluble and medium fractions) were imaged using the LI-COR Odyssey CLX Imaging System. The membranes with the insoluble and medium fractions were incubated with HRP-conjugated secondary antibodies diluted in Blot Incubation Buffer and were developed with Pico or Femto Chemiluminescent Substrate using the ImageQuant LAS 4000 system (GE Healthcare Life Sciences). All image analysis and quantifications were performed with the Image Studio Lite software.

### In-gel tryptic digestion and LC–MS/MS analysis

BG2-c6 cells were transiently transfected with the pMT-puro tau AP-3xHA plasmid in a 6-well plate using the Effectene Transfection Reagent (Qiagen) and according to the manufacturer’s instructions. Briefly, the cells were detached and for one well, 400 ng of plasmid were diluted in Buffer EC and 3.2 µl of the Enhancer was added. Subsequently, 10 µl of the Effectene Transfection Reagent was added, the reaction was incubated for 10 min at RT and complete medium was added up to 1000 µl. The complexes were mixed with an equal volume of cells at a final concentration of 1.5 10^6^ cells/ml and they were plated on concanavalin A (ConA, 0.5 mg/ml)-coated plates. The complexes were replaced with fresh medium after 6 h and 3 days later the cells were expanded in T75 flasks. At this stage the expression of tau AP-3xHA was induced by adding 1 mM CuSO_4_ for 3 days.

Subsequently, the cells were detached and lysed in Lysis Buffer for 30 min on ice. The lysate was clarified and subjected to immunoprecipitation using the Anti-HA Magnetic Beads (Thermo Fisher Scientific) according to the manufacturers’ instructions. The immunoprecipitated proteins were eluted with 30 µl of IP Elution Buffer and incubated for 10 min at RT while rotating. At the eluted fraction 7.5 µl of IP Neutralization Buffer) and 7.5 µl of 4 × Lämmli sample buffer were added (final volume 45 µl) and the mixture was boiled for 10 min at 95 °C. The eluted fractions wetre analyzed by SDS-PAGE and stained with colloidal Quick Coomassie stain (Serva). The Coomassie stained bands were cut out and gel slices were transferred to a 96-well plate, automatically reduced, alkylated, and digested with trypsin^85^. The peptides were isolated from the gel pieces with 50% acetonitrile/ 0.1% TFA, concentrated nearly to dryness in a SpeedVac vacuum centrifuge and diluted to a total volume of 30 µl with 0.1% TFA. 10 µl of the sample were analyzed by a Dionex UltiMate 3000RSLCnano HPLC system (Thermo Fisher Scientific) coupled either to a LTQ Orbitrap Elite or Q-Exactive-HF mass spectrometer (both Thermo Fisher Scientific). Samples analyzed on the LTQ Orbitrap Elite were loaded on a C18 trapping column (Acclaim PepMap100, C18, 5 µm, 300 µm i.d. × 5 mm, Thermo Fisher Scientific), using a loading buffer of 0.1% TFA at a flow rate of 30 µl/min. Peptides were eluted and separated on a C18 analytical column (Acclaim PepMap RSLC C18, 2 µm, 75 µm i.d. × 25 cm, Thermo Fisher Scientific) with a flow rate of 300 nl/min in a 50 min gradient from 96% MS Buffer A (1% acetonitrile, 0.1% formic acid) and 4% MS Buffer B (90% acetonitrile, 0.1% formic acid) to 60% MS Buffer A and 40% buffer B. One Orbitrap survey scan was followed by up to 20 data-dependent product ion scans in the LTQ ion trap. Samples analyzed on the Q-Exactive-HF system were directly injected to an analytical column (75 µm × 300 mm), which was self-packed with 3 µm Reprosil Pur-AQ C18 material (Dr. Maisch HPLC GmbH) and separated using a gradient from 97% MS Buffer A and 3% MS Buffer B to 40% MS Buffer A and 60% MS buffer B. MS data were acquired with an automatic switch between a full scan and up to 15 data-dependent MS/MS scans. To increase sensitivity for phosphorylated peptides of the target protein, inclusion lists consisting of m/z values for possible phosphorylated peptides were applied. The uninterpreted MS/MS spectra were searched against the target protein sequences, a reverse decoy version of these sequences and a common laboratory contaminant database, using the MaxQuant software with its built-in Andromeda search algorithm^86^. The algorithm was set to use trypsin as proteolytic enzyme allowing up to two missed cleavages, assuming carbamidomethylation as a fixed modification of cysteine, and oxidized methionine, acetylated N-termini and phosphorylation (serine, threonine, tyrosine) as variable modifications. All other parameters were set to default MaxQuant specifications. The false discovery rate (FDR), determined by searching the reverse database, was set at 0.01 for both peptides and proteins. Modified peptides had additionally to exceed an andromeda score of 40 and a delta score of 6.

## Supplementary Information

Supplementary Information.
