# Supplementary material for: Identification of cis-acting determinants mediating the unconventional secretion of tau
Source: Sci Rep. 2021 Jun 21;11:12946. doi: 10.1038/s41598-021-92433-3 (PMC8217235; doi:10.1038/s41598-021-92433-3)

Identification of *cis*-acting determinants mediating the unconventional secretion of tau

Taxiarchis Katsinelos[1,2,3], William A. McEwan[3], Thomas R. Jahn[2], Walter Nickel[1]

[1]Heidelberg University Biochemistry Center (BZH), INF 328, 69120 Heidelberg, Germany.
[2]Schaller Research Group at the University of Heidelberg and the DKFZ, Proteostasis in Neurodegenerative Disease (B180), INF 581, 69120 Heidelberg, Germany.
[3]Department of Clinical Neurosciences, UK Dementia Research Institute at the University of Cambridge, Cambridge, UK

Correspondence and requests for materials should be addressed to T.K. (tk537@cam.ac.uk)

Supplementary information

Figure S1

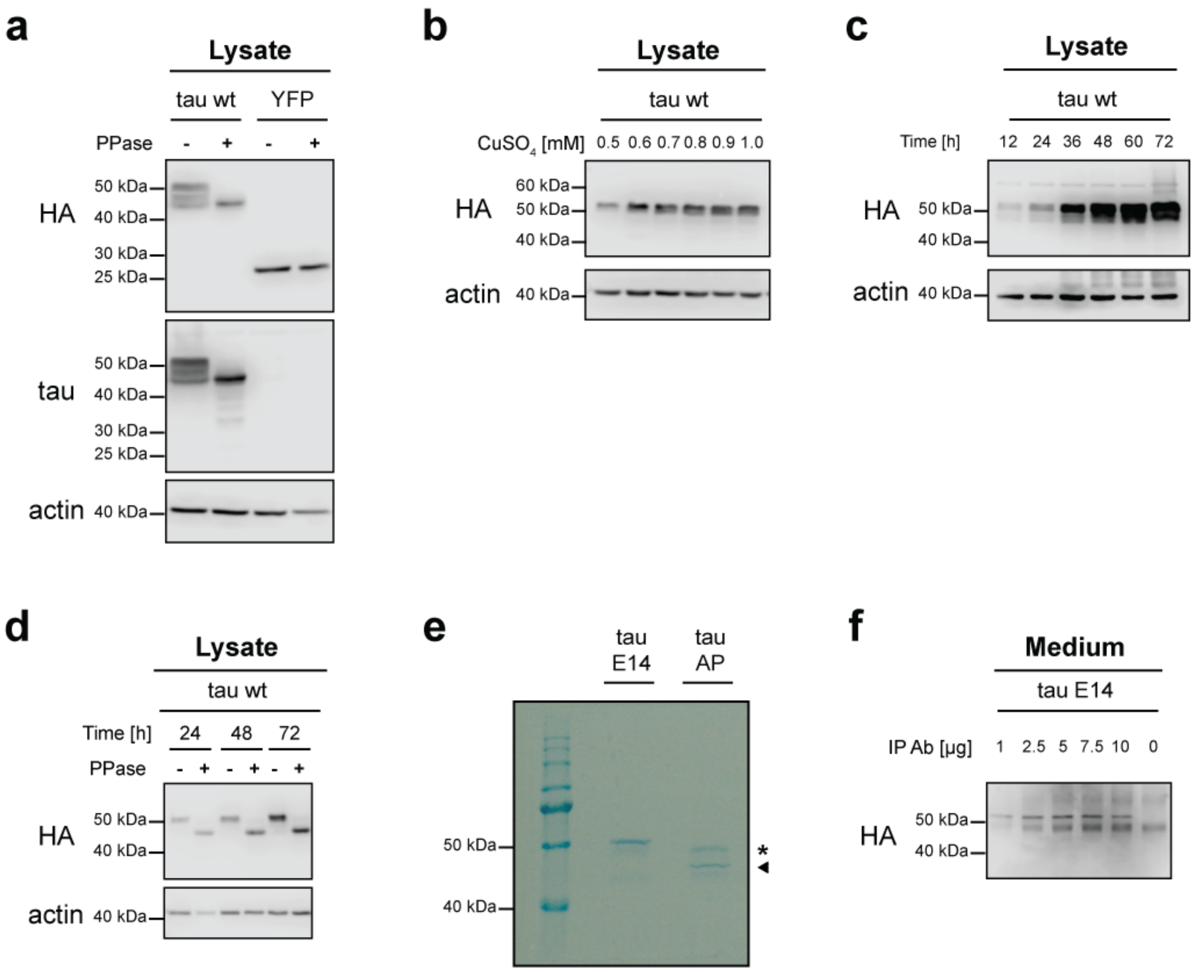

a
Lysate
tau wt
YFP
PPase
-
+
-
+
HA
50 kDa
40 kDa
30 kDa
25 kDa
tau
50 kDa
40 kDa
30 kDa
25 kDa
actin
40 kDa
b
Lysate
tau wt
CuSO4 [mM]
0.5 0.6 0.7 0.8 0.9 1.0
HA
60 kDa
50 kDa
40 kDa
actin
40 kDa
c
Lysate
tau wt
Time [h]
12 24 36 48 60 72
HA
50 kDa
40 kDa
actin
40 kDa
d
Lysate
tau wt
Time [h]
24 48 72
PPase
- + - + - +
HA
50 kDa
40 kDa
actin
40 kDa
e
tau
E14
tau
AP
50 kDa
40 kDa
*
f
Medium
tau E14
IP Ab [µg]
1 2.5 5 7.5 10 0
HA
50 kDa
40 kDa

Figure S1. Characterization of a novel *Drosophila* neuronal cell culture system for tau secretion.

(a) Lysates from BG2-c6 neuronal cells expressing C-terminally 3xHA-tagged versions of tau wt and YFP were dephosphorylated and then blotted against HA and pan-tau. Blotting against actin was used as loading control. **(b)** Increasing amounts of $CuSO_4$ were employed for inducing the expression of tau wt and the lysates were blotted against HA and actin. **(c)** Cells expressing tau wt were lysed at different time points and lysates were blotted against HA and actin. **(d)** Phosphatase-treated and untreated lysates from BG2-c6 cells expressing tau wt for different time intervals were blotted against HA and actin. **(e)** BG2-c6 cells expressing either tau E14 or tau AP were lysed and then subjected to immunoprecipitation. The eluted tau fractions were then analysed through SDS-PAGE and subsequently stained with Coomassie. The asterisk and arrow indications correspond to the P- and non-P band of tau AP, respectively. **(f)** Conditioned medium from BG2-c6 neuronal cells expressing tau E14 were equally distributed and subsequently subjected to immunoprecipitation with increasing amounts of the pan-tau KJ9A antibody. The eluted fractions were then immunoblotted using an antibody against the HA-tag.

Figure S2

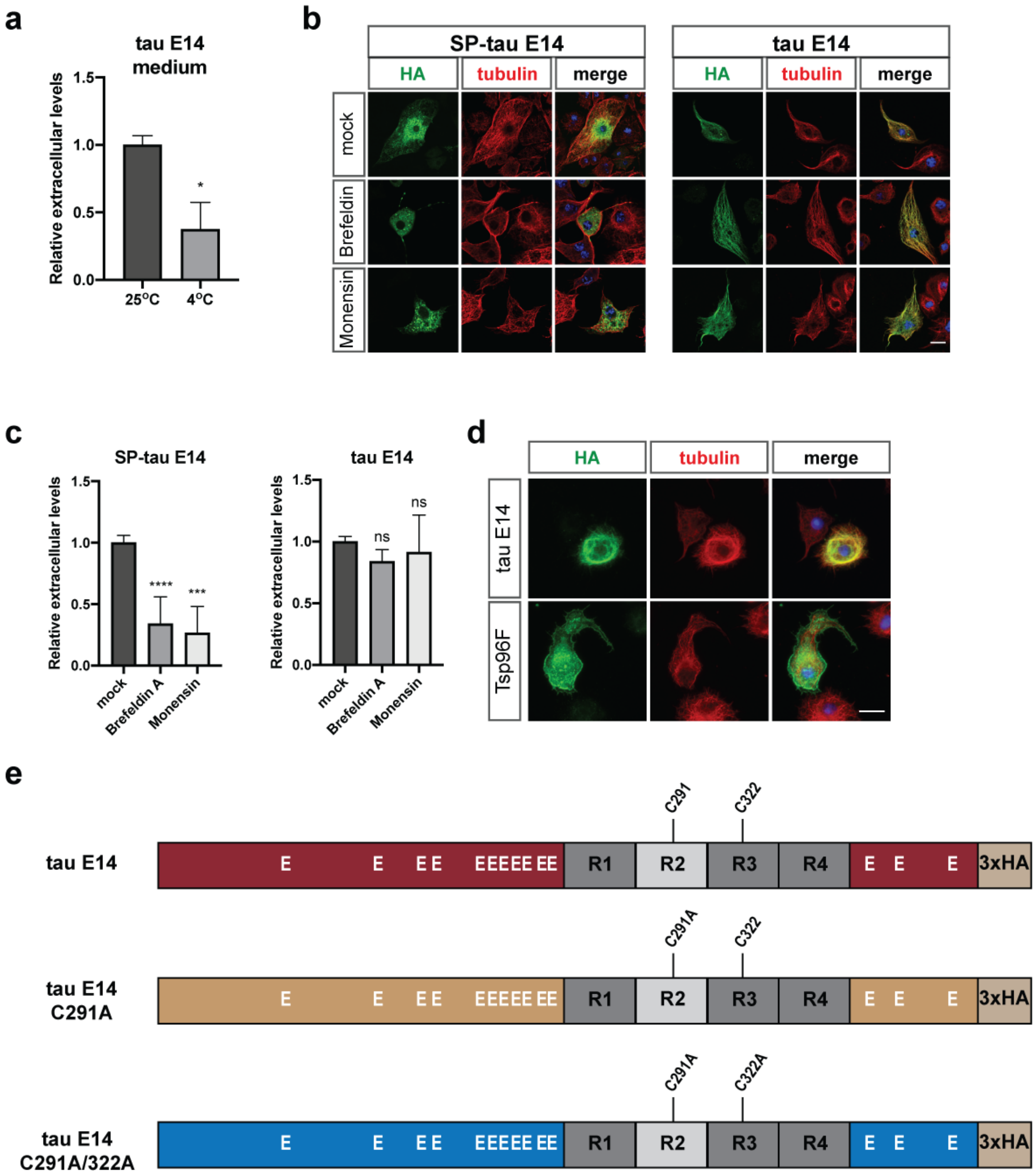

a
tau E14 medium
Relative extracellular levels
1.5
1.0
0.5
0.0
*
25°C
4°C
b
SP-tau E14
tau E14
HA
tubulin
merge
mock
Brefeldin
Monensin
c
SP-tau E14
tau E14
Relative extracellular levels
****
***
ns
ns
mock
Brefeldin A
Monensin
d
HA
tubulin
merge
tau E14
Tsp96F
e
tau E14
tau E14 C291A
tau E14 C291A/322A
C291
C322
C291A
C322A
E
R1
R2
R3
R4
3xHA

Figure S2. The secretion of tau is not related to the canonical ER/Golgi secretory pathway.

(**a**) The secreted amounts from cells cultured for 6 h either at 25 ^{o}C or at 4 ^{o}C were densitometrically quantified, normalized to the intracellular levels, and subsequently compared to the normal culturing conditions. The data represent mean values ± s.d. derived from n = 3 biological replicates and were subjected to unpaired t test that was followed by Welch's correction. **(b)** Cells transfected with SP-tau E14 and tau E14 were treated with Brefeldin A and Monensin for 6 h before fixation and immunofluorescence staining was performed for tau, tubulin, and nucleus with antibodies against HA, tubulin, and Hoechst dye respectively (scale bar, 15 μm). **(c)** The densitometrically quantified secreted protein levels for every condition were initially normalized to the intracellular amounts and subsequently compared to the mock treatment. The data represent mean values ± s.d. derived from at least 3 biological replicates and were subjected to one-way ANOVA, followed by Tukey's post hoc test. **(d)** HA and tubulin antibodies were employed for detection of tau E14 or Tsp96F and tubulin, respectively, whereas Hoechst was used for nuclear staining (scale bar, 15 μm). **(e)** Schematic illustration of the tau E14 and the cysteine mutant tau variants generated and employed in the study. The light gray colouring of R2 indicates the domain that is absent in the 3R tau isoforms.

Figure S3

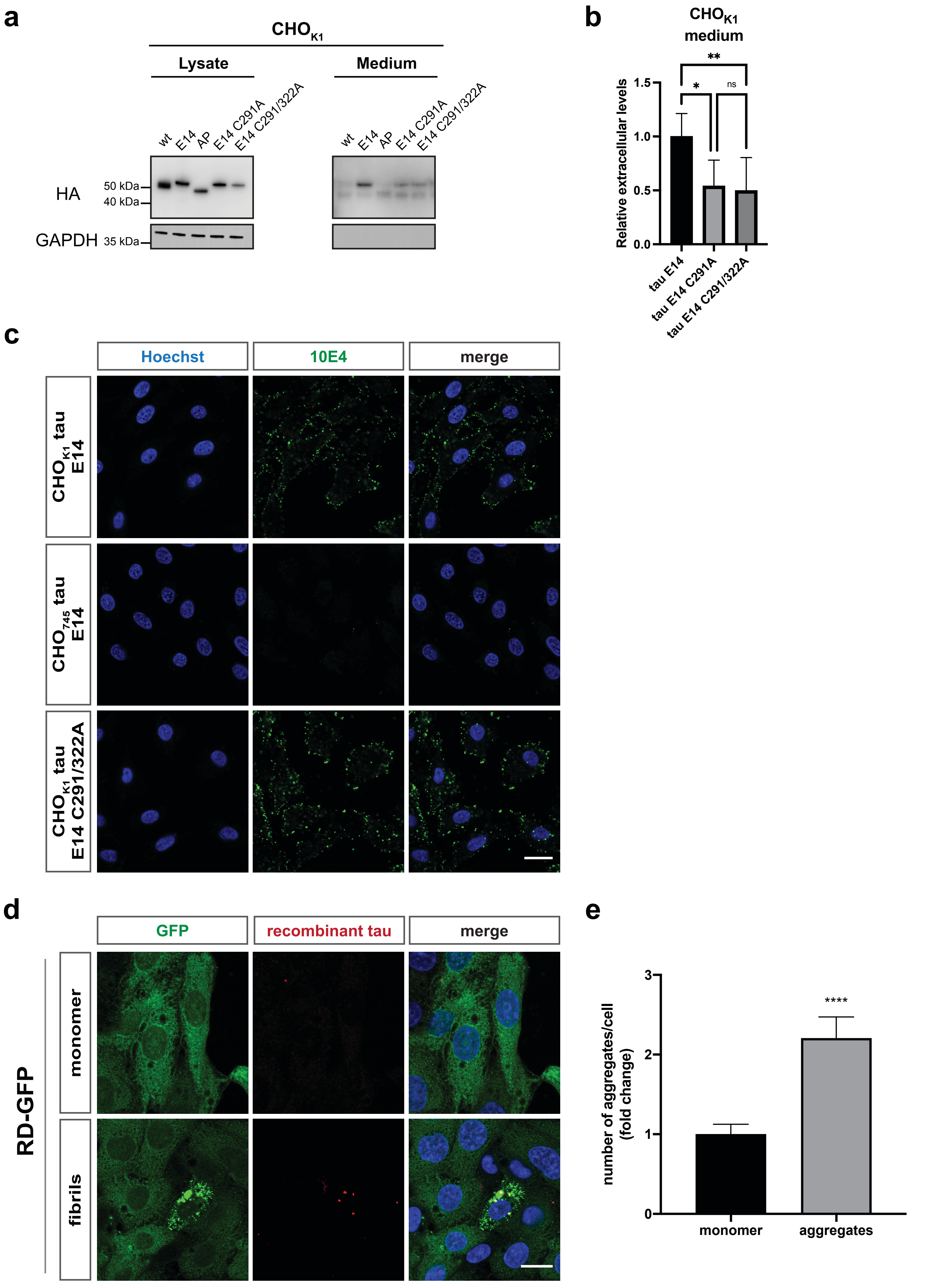
a
CHO K1
Lysate
Medium
wt
E14
AP
E14 C291A
E14 C291/322A
HA
50 kDa
40 kDa
GAPDH
35 kDa
b
CHO K1 medium
Relative extracellular levels
1.5
1.0
0.5
0.0
**
*
ns
tau E14
tau E14 C291A
tau E14 C291/322A
c
Hoechst
10E4
merge
CHO K1 tau E14
CHO 745 tau E14
CHO K1 tau E14 C291/322A
d
GFP
recombinant tau
merge
RD-GFP
monomer
fibrils
e
number of aggregates/cell (fold change)
3
2
1
0
****
monomer
aggregates

Figure S3. Cysteine mutants tau E14 expression in CHO_{K1} cells and validation of seeding competence in biosensor cell line.

(a) CHO_{K1} cells expressing the different variants were lysed and the conditioned medium was subjected to immunoprecipitation. Both fractions were immunoblotted against the HA-tag, while GAPDH was used as loading and quality control. **(b)** The densitometrically quantified secreted levels were initially normalized to the intracellular levels and subsequently compared to tau E14. The data represent mean values ± s.d. derived from at least 4 biological replicates and were statistically compered using one-way ANOVA, followed by Tukey's post hoc test. **(c)** Non-permeabilized fixed cells expressing tau E14 in normal and deficient sulfated proteoglycans background (CHO_{K1} and CHO_{745}) were compared to CHO_{K1} tau E14 C291/322A cells. The sulfated proteoglycans on the cell surface were stained using the monoclonal 10E4 antibody, whereas Hoechst was used for nuclear staining (scale bar, 15 µm). **(d)** CHO_{K1} cells expressing RD-GFP were transduced with monomeric or fibrillar tau E14 assemblies. The RD-GFP was stained using an anti-GFP antibody, while for the detection of the exogenously administered recombinant assemblies the tau10 antibody was deployed. Hoechst was used for staining of the nucleus (scale bar, 15 µm). **(e)** The GFP-positive inclusions were quantified using a semi-automated image-based analysis. The number of aggregates per cell for each condition were finally compared to the monomer-treated (3 independent biological replicates with at least 800 cells per case were processed). Data represent mean values ± SEM and were subjected to unpaired t test that was followed by Welch's correction.

Supplementary Table S1 Summary of phosphorylated epitopes for tau AP.

Table of all phospho-sites as detected using LC-MS/MS for the two bands of the tau AP variant with distinct mobility under denaturing SDS-PAGE conditions when expressed in BG2-c6 cells. The detailed lists of the identified peptides are presented in Tables 2 and 3. Epitopes with low probability of < 25% were excluded and the ones with a probability ≥ 25% were divided in two confidence groups (≥ 75% for high and 25% ≤ x < 75% for moderate confidence). The numbering of the epitopes is based on the longest (2N4R) human tau isoform. Epitopes marked in red correspond to sites that are phosphorylated in the high (p-band), but not in the lower molecular weight (low p-band) version of the tau AP variant.

	BG2-c6 cells	
	tau AP P-band	tau AP low P-band
Confidence ≥ 0.75	S113, S208, S214, S262, S285, S356, Y394, S400, T403	S113, S208, S214, S262, S285, S356,
0.25 ≤ Confidence < 0.75	S191, S195, Y197, S198, S210, T263, S412, T414, S416	S191, S195, Y197, S198, S210, S412, T414, S416

Supplementary Table S2 Tau AP p-band

The amino acids marked in red correspond to serine or threonine residues from the original tau wt variant that are mutated to alanine for the tau AP variant. Green marked epitopes correspond to oxidized residues, while yellow and grey marking (accompanied with site identification in italics) correspond to phosphorylated epitopes with high (probability ≥ 75%) and moderate confidence (25% ≤ probability < 75%), respectively.

Sequence	Site	Modification	m/z	Charge (z)	Mass (Da)
^{2}AEPRQEFEVMEDHAGTYGLGDR^{23}	M11	Oxidation (M)	855.715	3	2564.123
^{6}QEFEVMEDHAGTYGLGDR^{23}	M11	Oxidation (M)	690.634	3	2068.879
^{6}QEFEVMEDHAGTYGLGDRK^{24}	M11	Oxidation (M)	733.332	3	2196.974
^{24}KDQGGYTMHQDQEGDTDAGLK^{44}	M31	Oxidation (M)	770.669	3	2308.986
^{25}DQGGYTMHQDQEGDTDAGLK^{44}	M31	Oxidation (M)	727.971	3	2180.892
^{25}DQGGYTMHQDQEGDTDAGLK*AEEAGIGDAPSLEDE-AAGHVTQAR^{126}	M31	Oxidation (M)	912.205	5	4555.990
^{103}AEEAGIGDAPSLEDEAAGHVTQAR^{126}	S113	Phospho (STY)	825.365	3	2473.075
^{181}APPSSGEPPKSGDRSGYSAPGAPGAPGSR^{209}	*S191/S195/Y197/S198*	Phospho (STY)	721.655	3	2161.945
^{195}SGYSAPGAPGAPGSR^{209}	S208	Phospho (STY)	1024.861	2	2047.708
^{195}SGYSAPGAPGAPGSRSRAPSLPAPPTR^{221}	*S208/S210*, S214	2 Phospho (STY)	766.737	3	2297.189
^{210}SRAPSLPAPPTREPK^{224}	S214	Phospho (STY)	842.432	2	1682.851
^{212}APSLPAPPTREPK^{224}	S214	Phospho (STY)	480.913	3	1439.717
^{241}SRLQTAPVPMPDLK^{254}	M250	Oxidation (M)	784.926	2	1567.839
^{241}SRLQTAPVPMPDLKNVK^{257}	M250	Oxidation (M)	637.355	3	1909.045
^{243}LQTAPVPMPDLK^{254}	M250	Oxidation (M)	663.360	2	1324.706
^{243}LQTAPVPMPDLKNVK^{257}	M250	Oxidation (M)	556.311	3	1665.912
^{258}SKIGSTENLKHQPGGGK^{274}	S262	Phospho (STY)	637.356	3	1909.046
^{260}IGSTENLK^{267}	S262	Phospho (STY)	471.220	2	940.426
^{260}IGSTENLKHQPGGGK^{274}	S262	Phospho (STY)	534.926	3	1601.756
^{260}IGSTENLKHQPGGGKVQIINK^{280}	*S262/T263*	Phospho (STY)	766.737	3	2297.189
^{281}KLDLSNVQSK^{290}	S285	Phospho (STY)	401.720	4	1602.849
^{354}IGSLDNITHVPGGGNK^{369}	S356	Phospho (STY)	829.898	2	1657.782
^{354}IGSLDNITHVPGGGNKK^{370}	S356	Phospho (STY)	596.299	3	1785.877
^{384}AKTDHGAEIVYKAPVVSGDTAPR^{406}	Y394	Phospho (STY)	616.307	4	2461.200
^{384}AKTDHGAEIVYKAPVVSGDTAPR^{406}	T403	Phospho (STY)	616.307	4	2461.200

386TDHGAEIVYKAPVVSGDTSPR406	T403	Phospho (STY)	755.030	3	2262.068
396APVVSGDTAPR406	S400	Phospho (STY)	575.268	2	1148.523
396APVVSGDTAPR406	T403	Phospho (STY)	575.268	2	1148.523
407HLSNVSSTGSIDMVDAPQLATLADEVSASLAK438	M419/*S412/S413/T414/ S416*	Phospho (STY)	1135.184	3	3402.530

Supplementary Table S3 Tau AP non(p)-band

The amino acids marked in red correspond to serine or threonine residues from the original tau wt variant that are mutated to alanine for the tau AP variant. Green marked epitopes correspond to oxidized residues, while yellow and grey marking (accompanied with site identification in italics) correspond to phosphorylated epitopes with high (probability ≥ 75%) and moderate confidence (25% ≤ probability < 75%), respectively.

Sequence	Site	Modification	m/z	Charge (z)	Mass (Da)
^{2}AEPRQEFEVMEDHAGTYGLGDR^{23}	M11	Oxidation (M)	855.715	3	2564.123
^{6}QEFEVMEDHAGTYGLGDR^{23}	M11	Oxidation (M)	690.634	3	2068.879
^{6}QEFEVMEDHAGTYGLGDRK^{24}	M11	Oxidation (M)	733.332	3	2196.974
^{24}KDQGGYTMHQDQEGDTDAGLK^{44}	M31	Oxidation (M)	578.253	4	2308.986
^{25}DQGGYTMHQDQEGDTDAGLK^{44}	M31	Oxidation (M)	727.971	3	2180.892
^{25}DQGGYTMHQDQEGDTDAGLK*AEEAGIGDAPSLEDE-AAGHVTQAR^{126}	M31	Oxidation (M)	912.205	5	4555.990
^{103}AEEAGIGDAPSLEDEAAGHVTQAR^{126}	S113	Phospho (STY)	825.365	3	2473.075
^{181}APPSSGEPPKSGDRSGYSAPGAPGAPGSR^{209}	*S191/S195/Y197/S198*	Phospho (STY)	721.655	3	2161.945
^{195}SGYSAPGAPGAPGSR^{209}	S208	Phospho (STY)	1024.861	2	2047.708
^{195}SGYSAPGAPGAPGSRSRAPSLPAPPTR^{221}	*S208/S210*, S214	2 Phospho (STY)	766.737	3	2297.189
^{210}SRAPSLPAPPTREPK^{224}	S210	Phospho (STY)	842.432	2	1682.851
^{210}SRAPSLPAPPTREPK^{224}	S214	Phospho (STY)	842.432	2	1682.851
^{212}APSLPAPPTREPK^{224}	S214	Phospho (STY)	480.913	3	1439.717
^{241}SRLQTAPVPMPDLK^{254}	M250	Oxidation (M)	784.926	2	1567.839
^{241}SRLQTAPVPMPDLKNVK^{257}	M250	Oxidation (M)	637.355	3	1909.045
^{243}LQTAPVPMPDLK^{254}	M250	Oxidation (M)	663.360	2	1324.706
^{243}LQTAPVPMPDLKNVK^{257}	M250	Oxidation (M)	556.311	3	1665.912
^{258}SKIGSTENLKHQPGGGK^{274}	S262	Phospho (STY)			
^{260}IGSTENLK^{267}	S262	Phospho (STY)	471.220	2	940.426
^{260}IGSTENLKHQPGGGK^{274}	S262	Phospho (STY)	766.737	3	2297.189
^{281}KLDLSNVQSK^{290}	S285	Phospho (STY)			
^{354}IGSLDNITHVPGGGNK^{369}	S356	Phospho (STY)	829.898	2	1657.782
^{354}IGSLDNITHVPGGGNKK^{370}	S356	Phospho (STY)	596.299	3	1785.877
^{407}HLSNVSSTGSIDMVDAPQLATLADEVSASLAK^{438}	M419/*S412/S413/T414/S416*	Phospho (STY)	1135.184	3	3402.530

Figure 1 Raw WB images

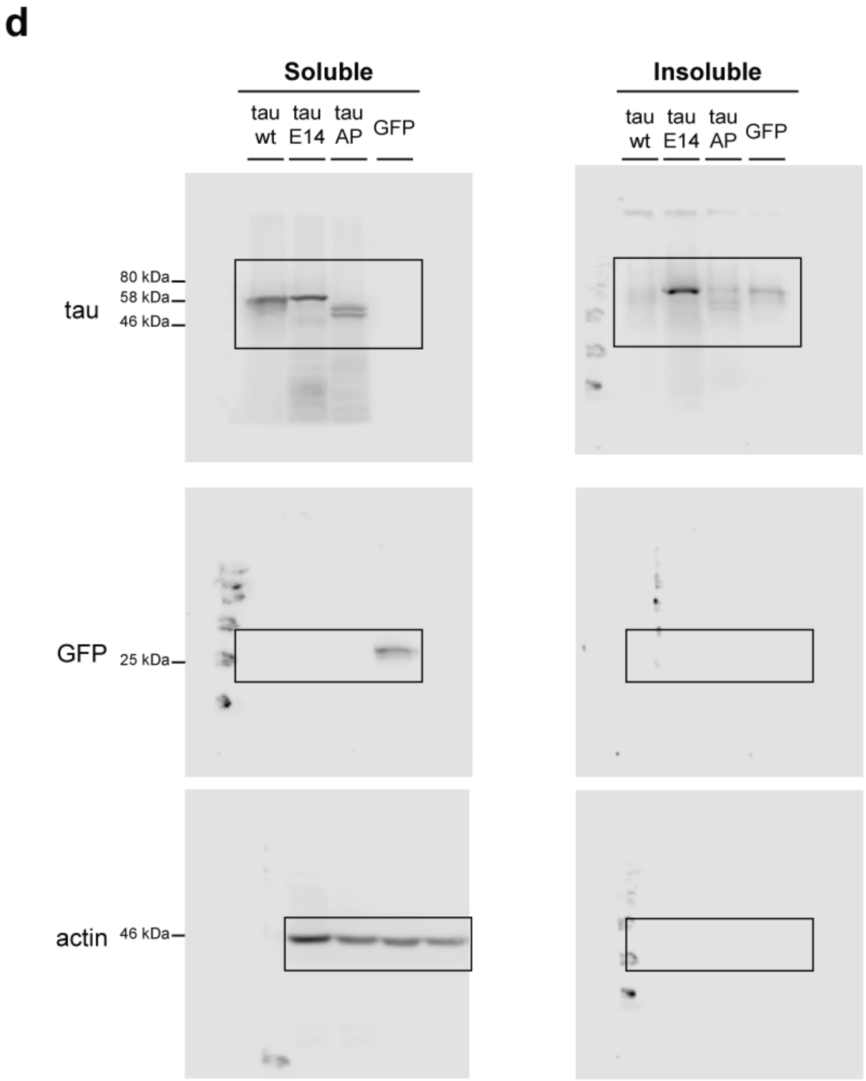

Figure 2 Raw WB images Part 1

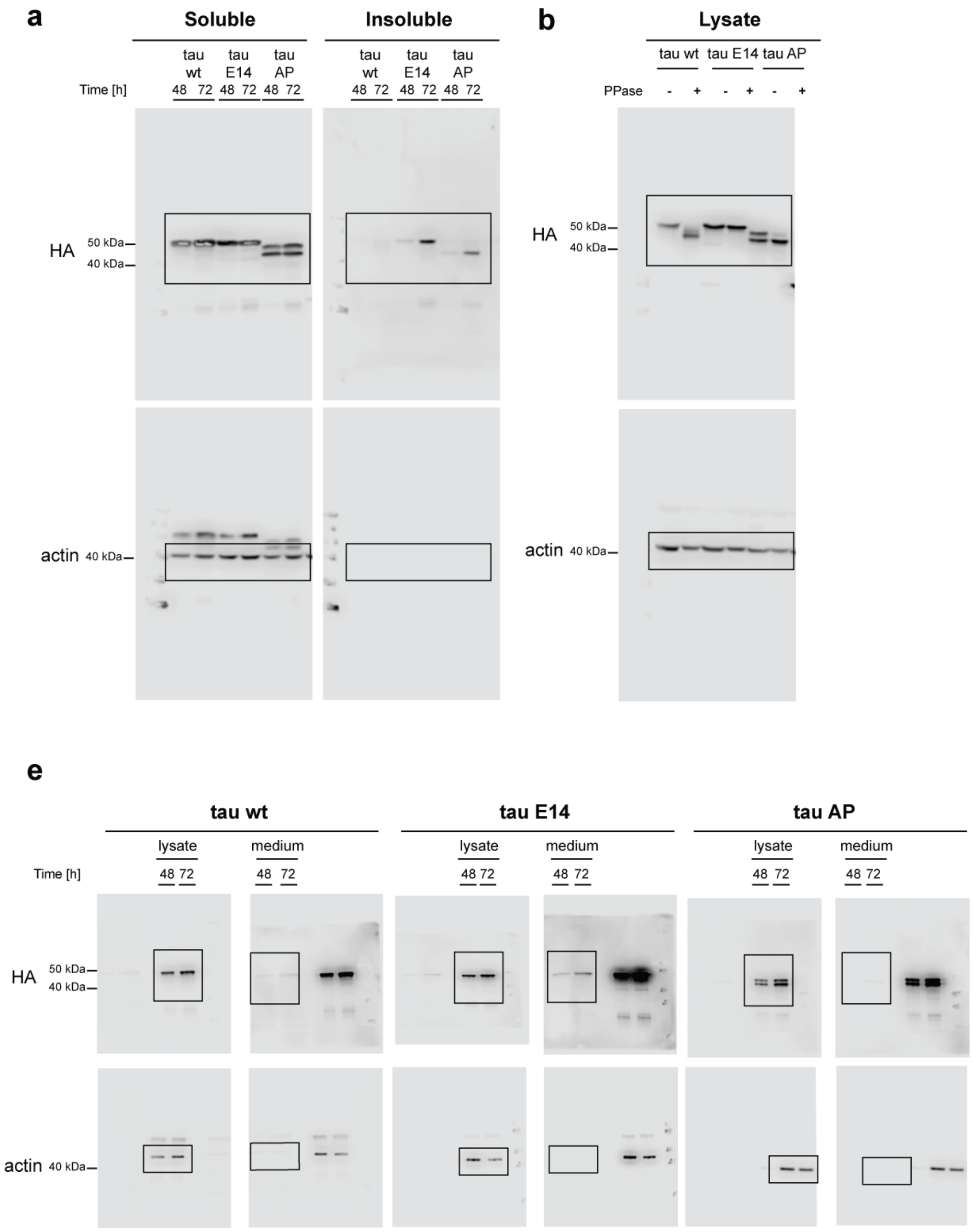

Figure 2 Raw WB images
Multiple exposures

e

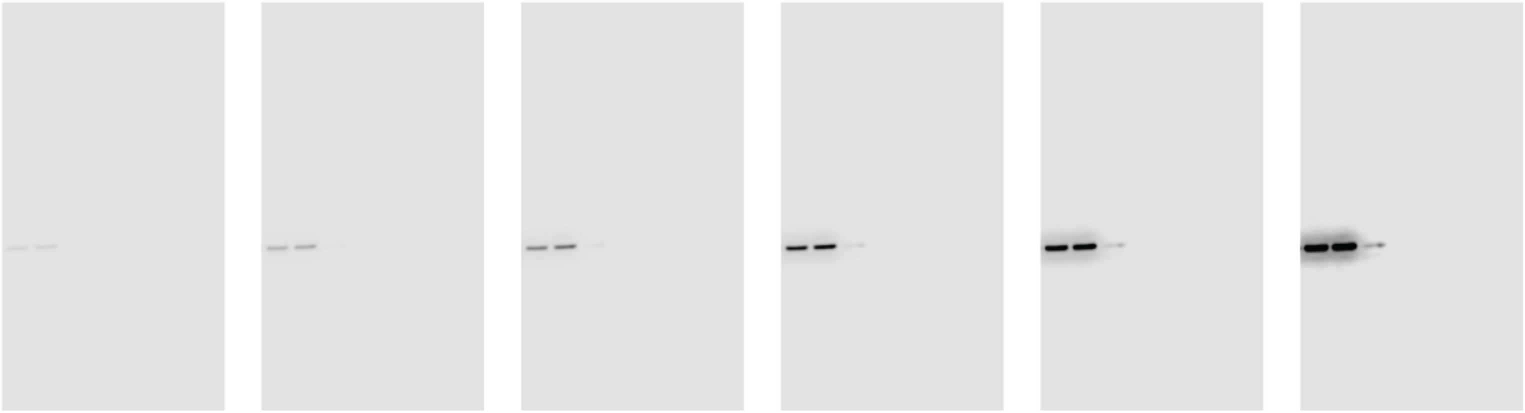

Figure 3 Raw WB images Part 1

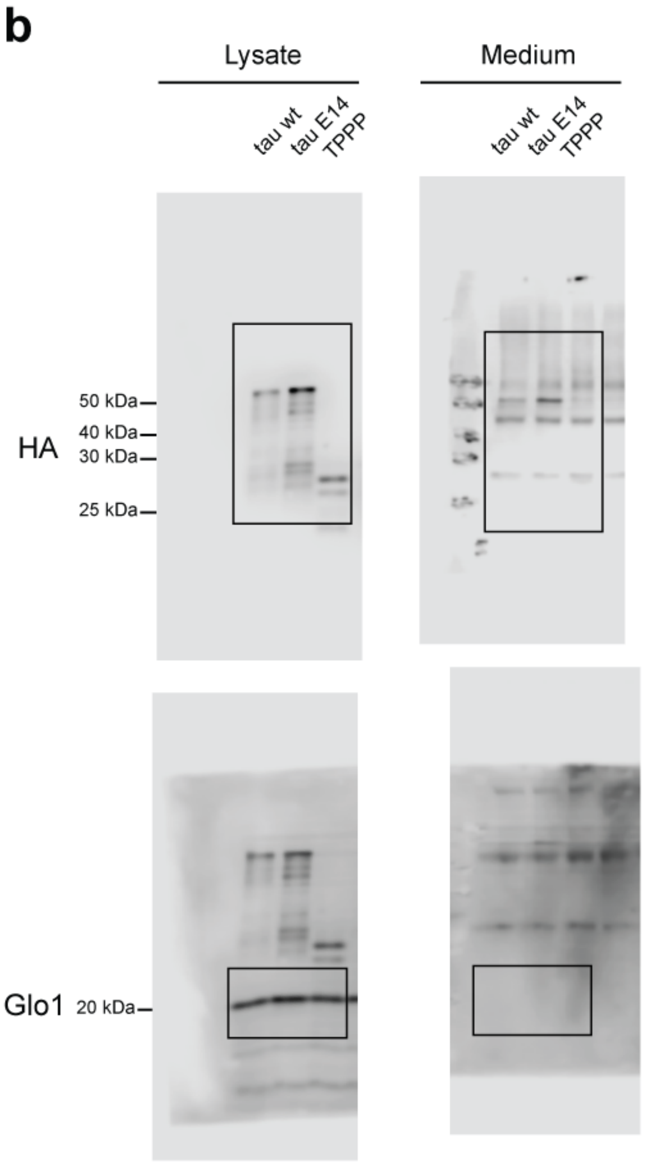

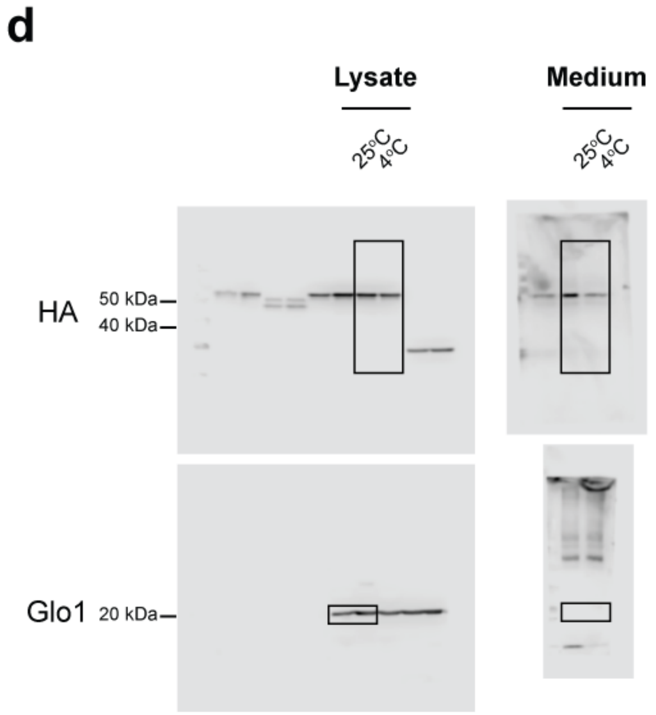

Figure 3 Raw WB images Part 2

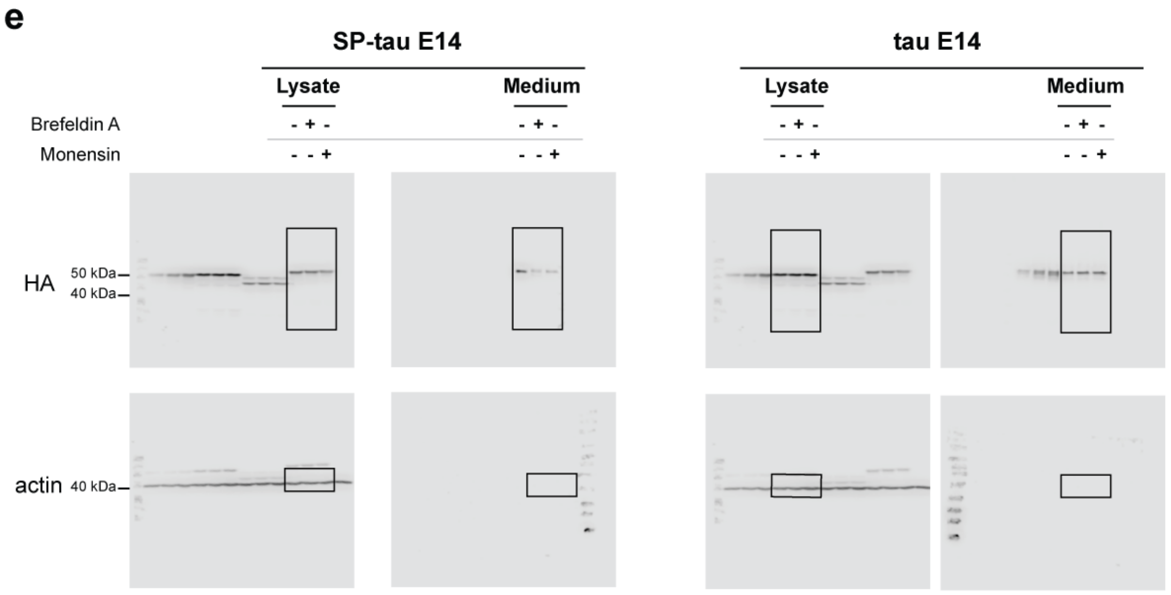

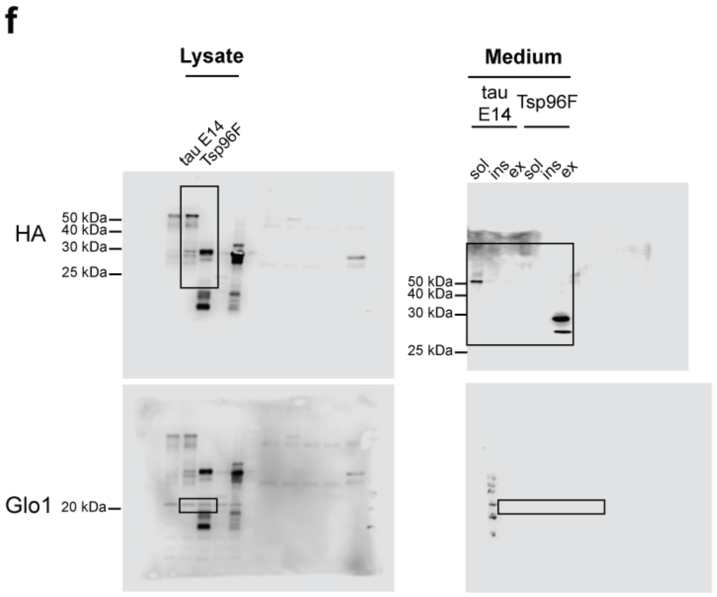

Figure 3 Raw WB images Part 3
Multiple exposures

f

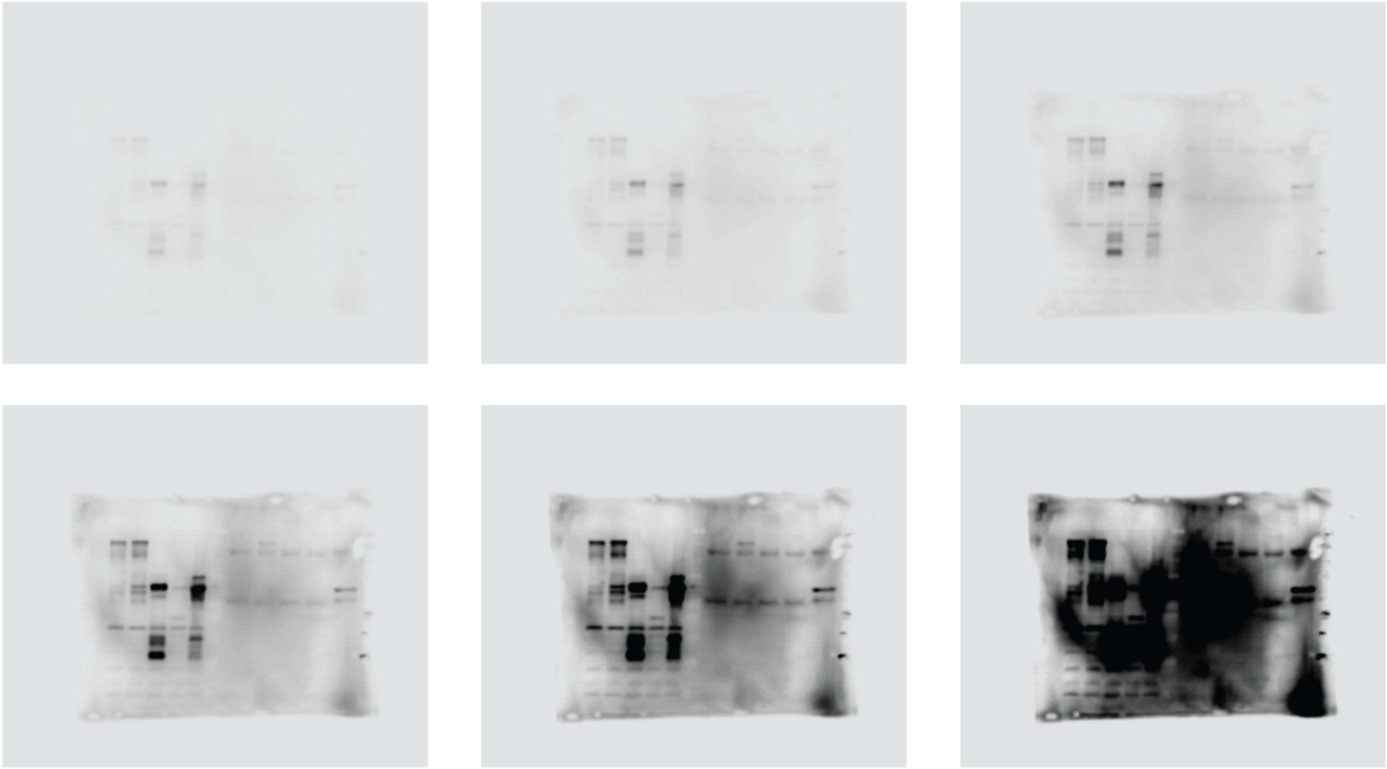

Figure 4 Raw WB images

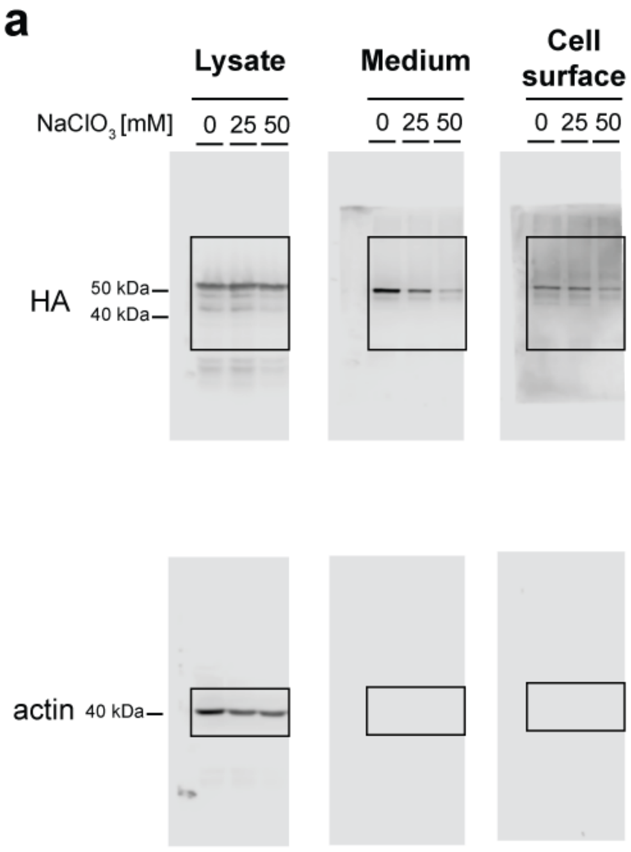

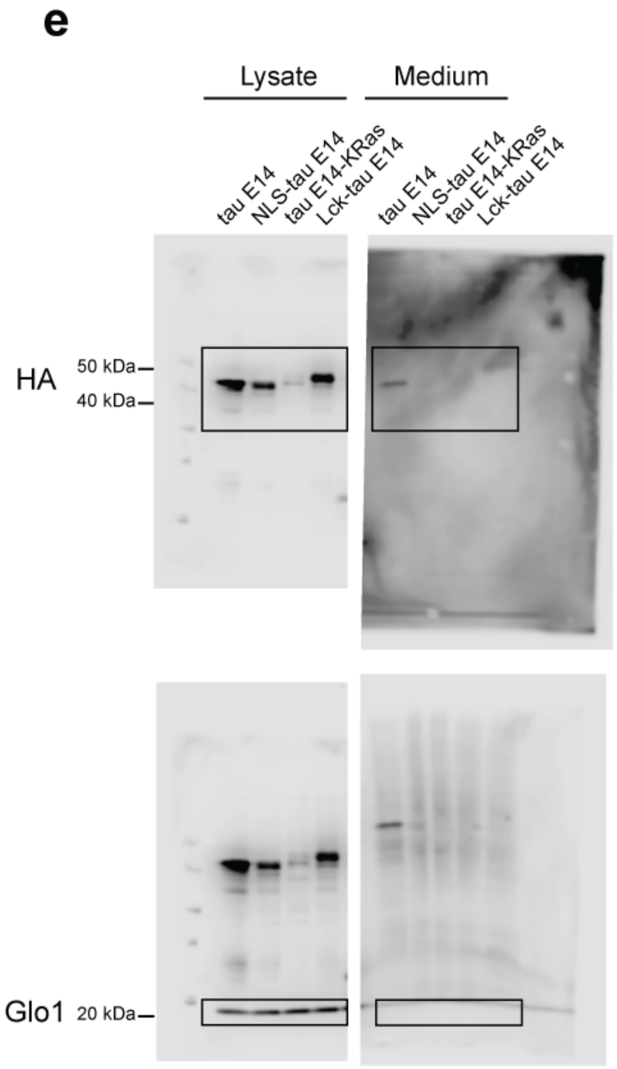

Figure 4 Raw WB images
Multiple exposures

a

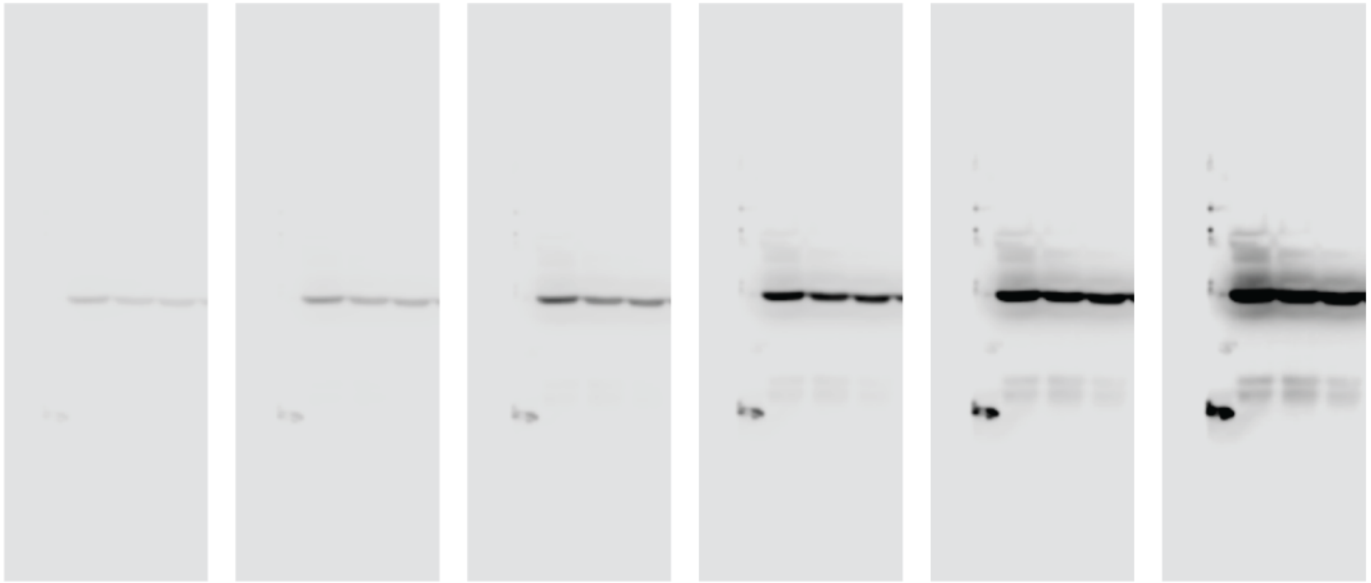

Figure 5 Raw WB images

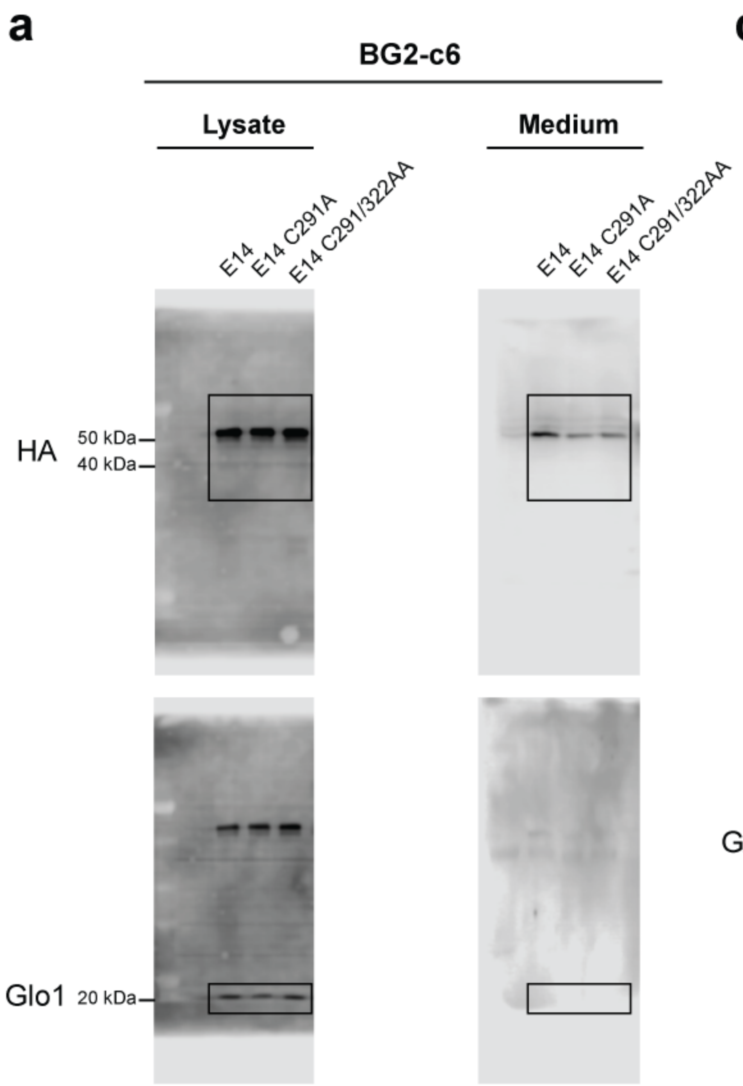

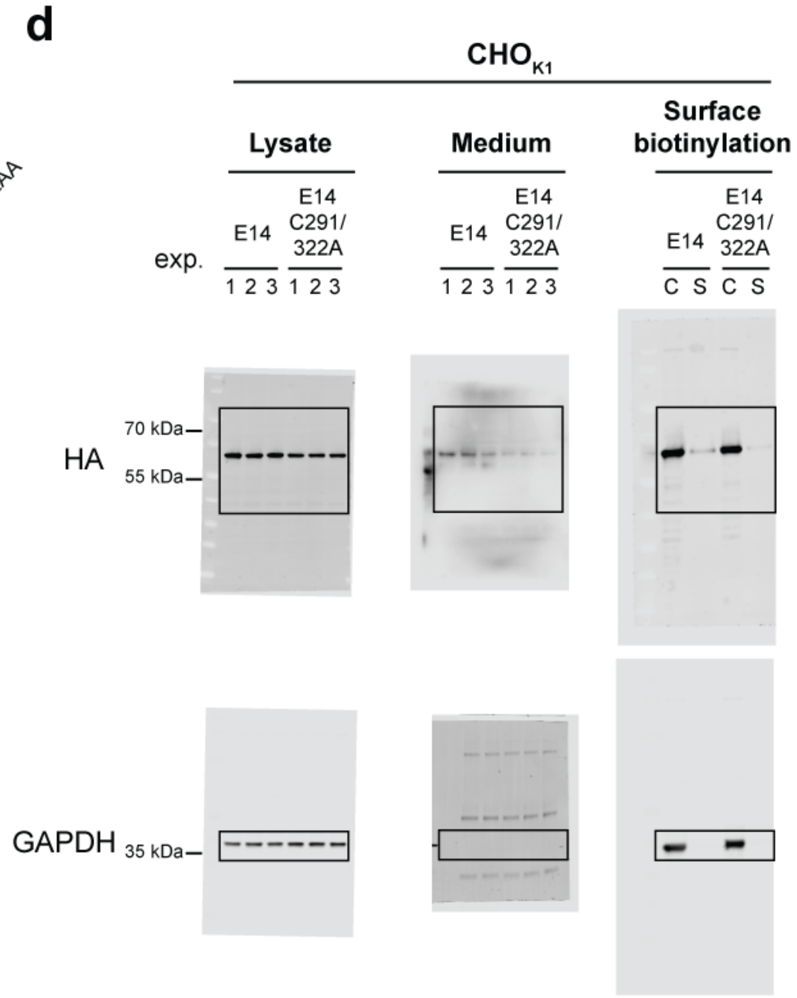

Figure S1 Raw WB images Part 1

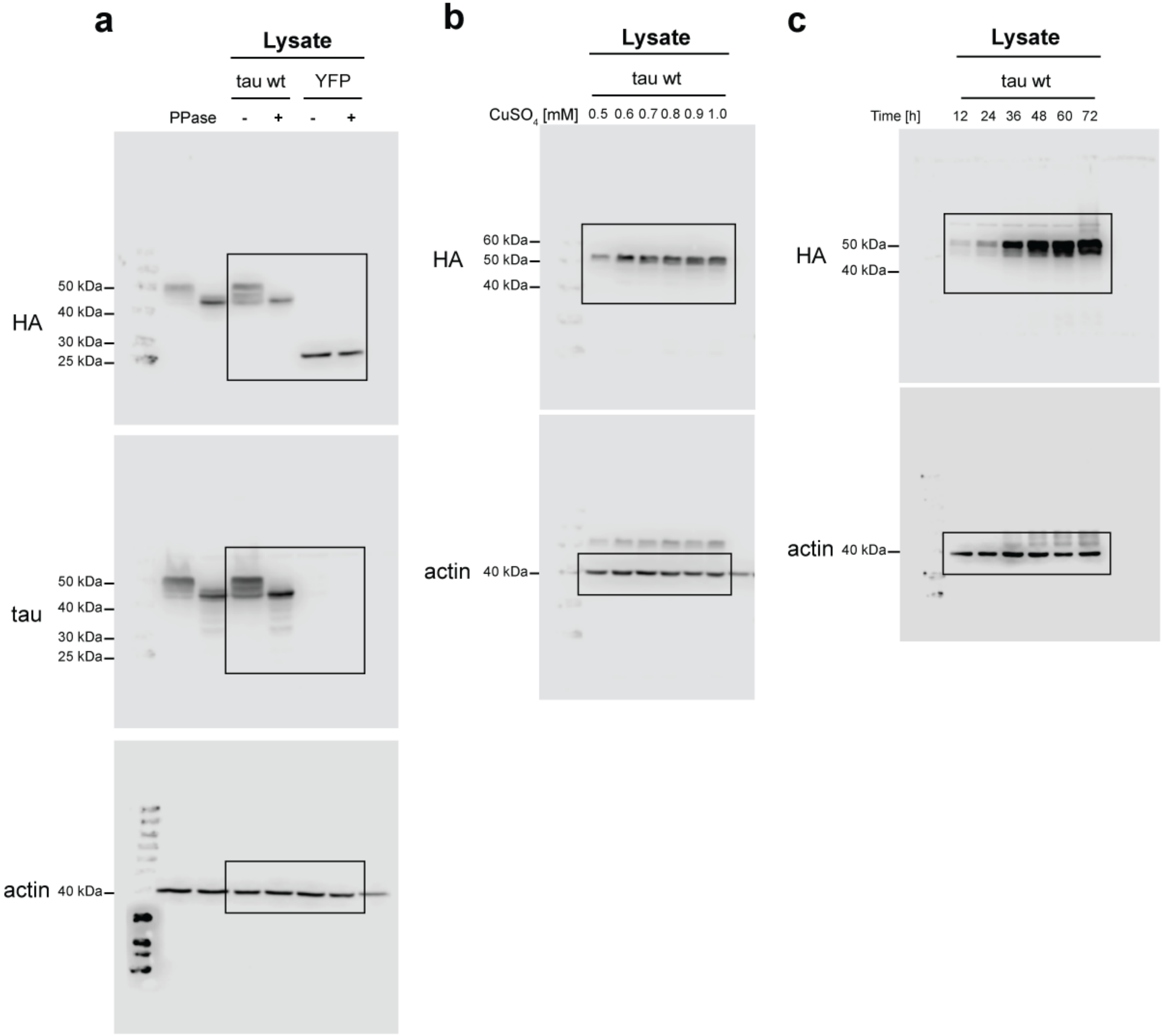

Figure S1 Raw WB images Part 2

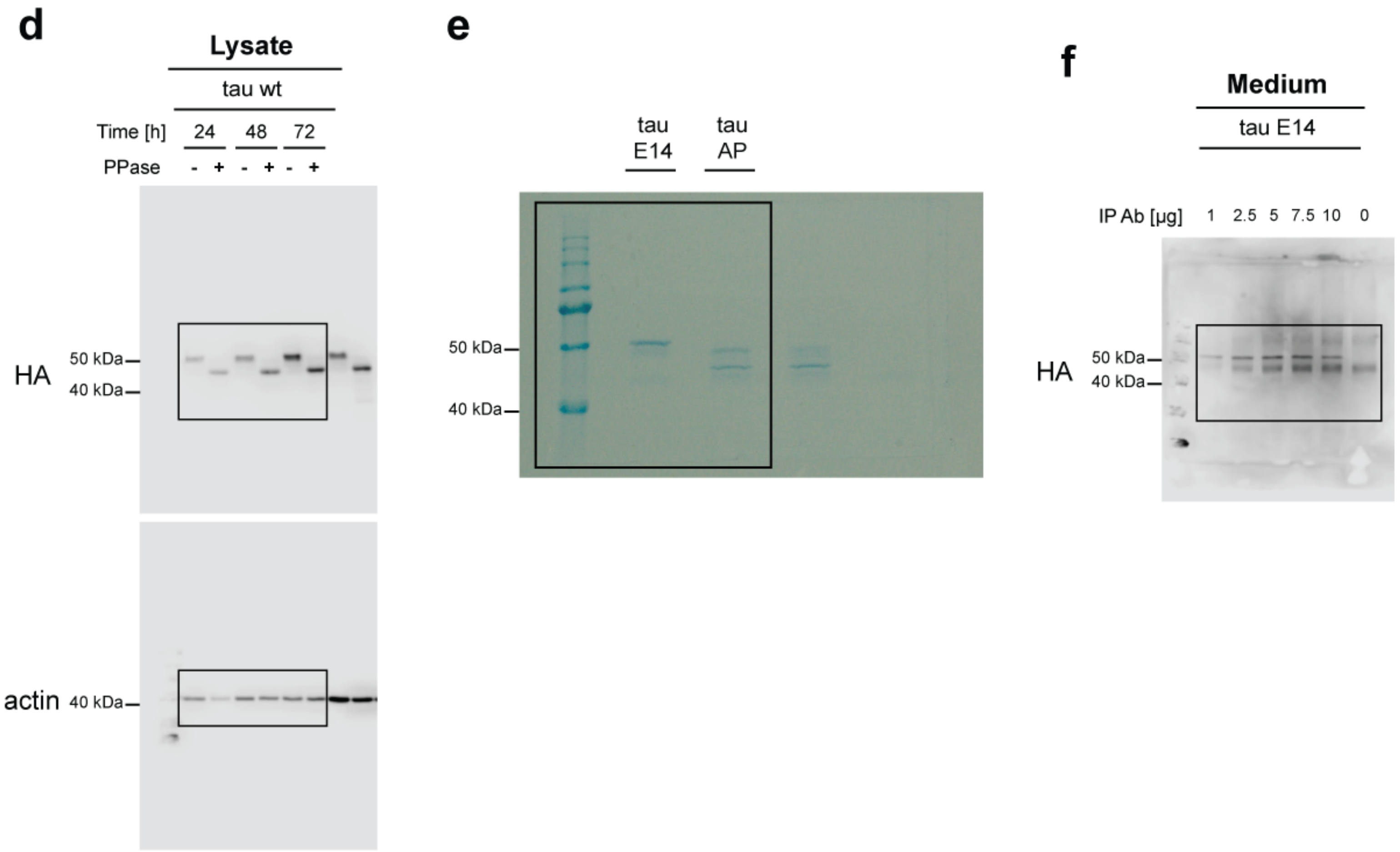

Figure S1 Raw WB images Part 3
Multiple exposures

d

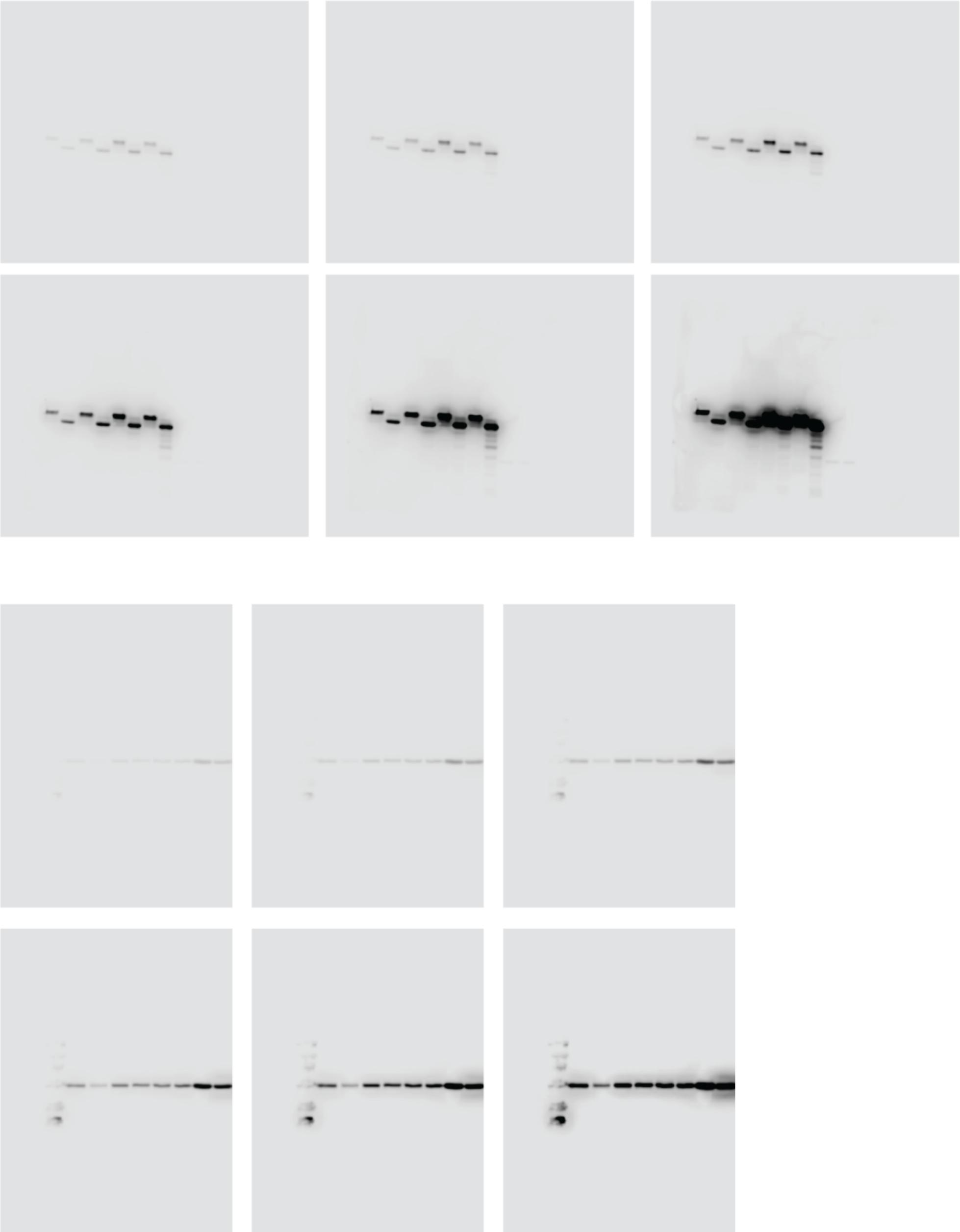

Figure S3 Raw WB images

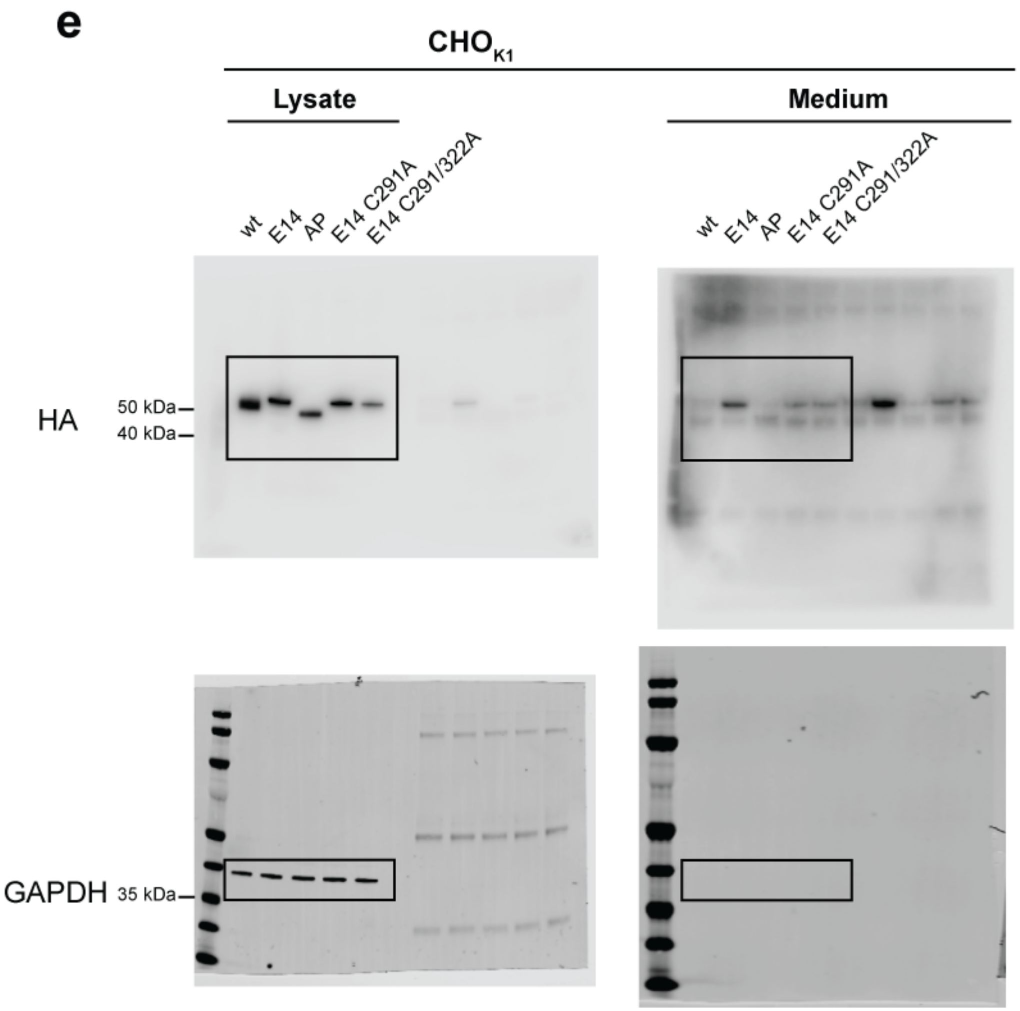

Supplement: Supplementary file 1 — Supplementary Information. [file 41598_2021_92433_MOESM1_ESM.pdf]
